# The Impact of Forest Thinning on the Reliability of Water Supply in Central Arizona

**DOI:** 10.1371/journal.pone.0121596

**Published:** 2015-04-02

**Authors:** Silvio Simonit, John P. Connors, James Yoo, Ann Kinzig, Charles Perrings

**Affiliations:** 1 EcoServices Group, School of Life Sciences, Arizona State University, Tempe, United States of America; 2 International Union for Conservation of Nature (IUCN), Regional Office for Mexico, Central America and the Caribbean, San Josè, Costa Rica; 3 School of Geographical Sciences and Urban Planning, Arizona State University, Tempe, United States of America; 4 California Baptist University, Riverside, CA, United States of America; University of California Davis, UNITED STATES

## Abstract

Economic growth in Central Arizona, as in other semiarid systems characterized by low and variable rainfall, has historically depended on the effectiveness of strategies to manage water supply risks. Traditionally, the management of supply risks includes three elements: hard infrastructures, landscape management within the watershed, and a supporting set of institutions of which water markets are frequently the most important. In this paper we model the interactions between these elements. A forest restoration initiative in Central Arizona (the Four Forest Restoration Initiative, or 4FRI) will result in thinning of ponderosa pine forests in the upper watershed, with potential implications for both sedimentation rates and water delivery to reservoirs. Specifically, we model the net effect of ponderosa pine forest thinning across the Salt and Verde River watersheds on the reliability and cost of water supply to the Phoenix metropolitan area. We conclude that the sediment impacts of forest thinning (up to 50% of canopy cover) are unlikely to compromise the reliability of the reservoir system while thinning has the potential to increase annual water supply by 8%. This represents an estimated net present value of surface water storage of $104 million, considering both water consumption and hydropower generation.

## Introduction

At the close of the first decade of this century, a special issue of the Proceedings of the National Academy of Sciences painted a grim picture of the sustainability of future water supplies in the American Southwest [1]. There is agreement among the general circulation models used to project global climatic change that the region will experience increased aridity as a result of a northerly expansion of the subtropical dry zones. Specifically, the region is expected to experience declining winter precipitation and increasingly severe and prolonged droughts over the 21st century. During this period, drought episodes (typified by continuous soil moisture depletion) are expected to increase from 4–10 years to periods of 12 years or more [2, 3]. At the same time, continued population growth is expected to increase demand for water, leading to increasing calls on surface and groundwater supplies. Humans now appropriate around 76% of streamflow in the region and it is estimated that a doubling of the population would increase this figure to 86% [4]. At the 2010 census, the Phoenix metropolitan area had a population of 4.2 million, and was growing at an annual rate of 2.7%. If that rate were to be maintained, the population would be over 7 million by 2030. Official population projections for Maricopa county are more cautious at 2.1% [5], but still imply a very significant increase over the next fifteen years. A study of the impact of population driven demand for water in the Phoenix Metropolitan Area of Arizona concluded that even if official projections of future population growth were halved, under most water supply scenarios current per capita water usage could not be sustained over the next decades while achieving groundwater "safe yields" (i.e. no net drawdown of the aquifer) by 2025 as established in the provisions of the Groundwater Management Act [6].

**Fig 1:**
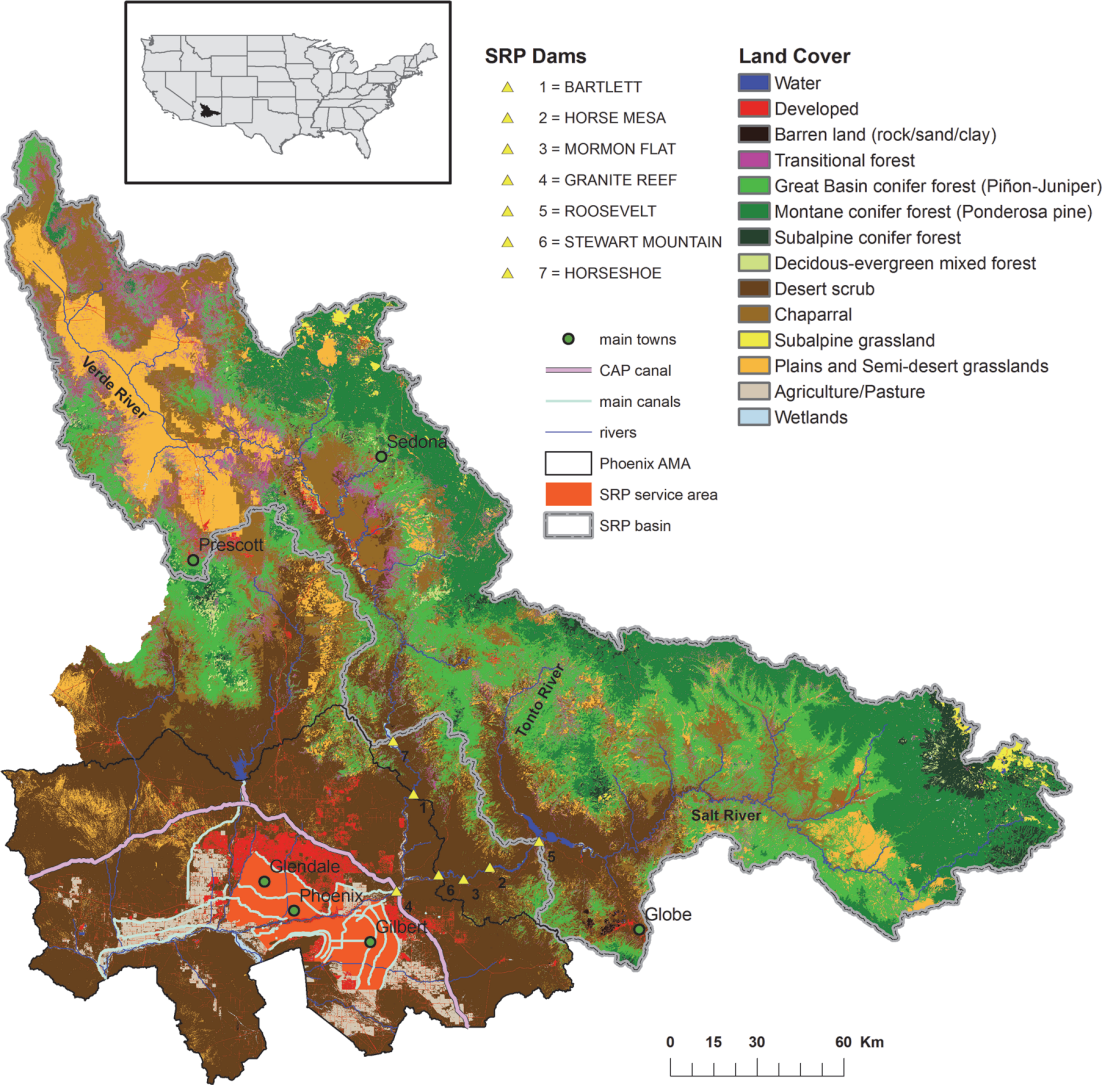
The area of study: Salt River Project (SRP) basin and SRP service area.

Economic growth in Central Arizona, as in other semiarid systems characterized by low and variable rainfall, has historically depended on the effectiveness of strategies to manage water supply and the risks of shortfall. These strategies have centered on the development of hard infrastructures (e.g., wells, canals, reservoirs), along with supporting institutions (e.g., water rights, water laws and water markets) to allocate available supplies among competing uses. The Phoenix metropolitan area depends on three main sources of water (Fig. 1): groundwater secured from the Phoenix aquifer within the Phoenix Active Management Area (AMA) where pumping is regulated to prevent overdraft, surface water from the Salt and Verde Rivers, and water from the Colorado River supplied through the Central Arizona Project. In this paper we focus on the Salt and Verde Rivers watersheds, and the water infrastructure operated by the Salt River Project (SRP). The SRP system comprises six reservoirs (Fig. 1 and Table 1) (four on the Salt and two on the Verde River) with a storage capacity of approximately 2.8 billion m^3^, plus a diversion dam at the confluence of the two rivers. The latter directs the flow into a 2,092 km long network of canals and laterals supplying water to a large segment of the Phoenix metropolitan area. The SRP service area comprises about 100,000 ha of agricultural and urban land (Fig. 1). SRP operates 248 wells within the service area, the aquifer being treated as an additional reservoir in the system. The aquifer is subject to a maximum annual pumping capacity from SRP wells of about 400 million m^3^. SRP delivers annually between 1.02 and 1.19 billion m^3^ of combined surface and groundwater to its shareholders and contract holders [7]. This represents between 37% and 43% of annual water demand in the Phoenix AMA [8].

**Table 1:** Salt River Project (SRP) storage capacity.

Dam	River	Reservoir name	Storage capacity*		Estimated water evaporation loss net of direct precipitation on lake surface
			(AF)	(million m^3^)	(million m^3^ year^-1^)
Roosevelt	Salt	Roosevelt Lake	1,653,043	2,039	59.77
Horse Mesa	Salt	Apache Lake	245,138	302	7.39
Mormon Flat	Salt	Canyon Lake	57,852	71	2.64
Stewart Mountain	Salt	Saguaro Lake	69,765	86	3.52
Horseshoe	Verde	Horseshoe Reservoir	109,217	135	7.57
Bartlett	Verde	Bartlett Reservoir	178,186	220	7.83
Granite Reef Diversion	Salt/Verde	-	-	-	-
*Total*			*2*,*313*,*201*	*2*,*853*	*88*.*71*

Note: *before adjusting for net evaporation.

In many systems, while hard water infrastructures of this sort have reduced vulnerability to variance in stream flows they have also made the system vulnerable to a new set of stressors. Reservoirs, for instance, are vulnerable to the effects of sedimentation. To adequately characterize the water supply system and its impact on water supply risks, we therefore need to include the features of the watershed—the environmental infrastructure—that affect risk. Both water and sediment yields are sensitive to land use and land cover (LULC) in watersheds. Land covers that increase runoff also tend to increase erosion and hence sedimentation. As a result, infrastructure-based strategies for the management of water risks in arid and semiarid areas require that the effects of land cover on water and sediment yields be balanced. While data on sedimentation of reservoirs in Arizona are sparse (there has only been one sediment survey in Roosevelt Lake on the Salt River, and none in any reservoir on the Verde River), it is believed that changes in land use and land cover in the two watersheds have at least the potential to affect sediment yields and hence the capacity of the reservoirs.

The selection of land cover in watersheds involves both coarse choices between vegetation types—grassland, forest and so on—and selection within vegetation types. It is now well established that forested catchments involve higher evapotranspiration rates than grassed catchments. So forested catchments would generally be expected to offer lower water yields than grassed catchments, but greater sediment control [9–12]. Forests optimized for water flows through canopy reduction, for example may be vulnerable to soil erosion. Given the large environmental and economic costs of restoring storage capacity, there has in recent years been a shift in paradigm toward managing existing dam projects as renewable resources through sediment control via soil conservation [13]. This builds on a long held view that soil conservation is amongst the most important sediment control strategies [14, 15]. The value of erosion control, one of the watershed regulating services recognized in the Millennium Ecosystem Assessment, partly lies in the protection of reservoir storage capacity. This in turns helps regulate the effect of inter-annual variability of water flows on the reliability of water supply.

In this paper we consider the trade-offs involved in managing the reliability of water flows and storage capacity through the modification of land cover in the Salt and Verde watersheds of Central Arizona. This extends recent efforts to model ecosystem service flows at the landscape scale [16–19]. By integrating spatially explicit hydrological and sediment transport models with models of ground and surface water supply and demand, we seek to derive the value of land cover change within the watersheds. To ensure that a reservoir system is able to meet water demand over the expected range of climatic conditions the feedbacks between watershed management, water infrastructures and water institutions should be explicitly addressed. Land use and land cover change in a watershed affects a wide range of ecosystem processes and functions aside from water and sediment supply depending on patterns of land ownership, market structures, local zoning and other land use restrictions. Since the value of any ecosystem service derives from the benefits that service offers to people [20], the value of the spatial distribution of land use/land cover across the basin derives from the value of the water and other ecosystem services the watershed delivers. This is seldom reflected in the market price of land, water or other commercially valuable watershed services. Rather, it is an ‘external’ cost or benefit of land cover change that affects people other than those responsible for that change [21, 22]. Moreover, the price paid for water itself seldom reflects the social opportunity cost of the resource. Water markets in the American west are the product of a long history of political negotiation and compromise that severely constrains their efficiency as allocation mechanisms [23, 24]. The demand for water is likewise the product of a history of economic development that reflects strategic decisions to promote or curtail particular sectors, interests and social groups [25, 26]. This makes the management of water risk not just a hydrological or an ecological problem, but also a political and economic problem.

Watershed management is not only linked with water flow regulation. The drought that affected the Southwest in the first decade of this century produced a sharp increase in the incidence of severe forest fires and pest infestations (bark beetles) [27]. While this partly reflected the effect of higher temperatures, earlier spring warming, and decreased surface water [1, 28], it also reflected a long recognized impact of fire suppression policy on forest density [29]. In response to the accumulated evidence of the effects of post-settlement forest management, the US Forest Service is undertaking a restoration programme in the ponderosa pine forest in the Salt and Verde watershed as part of the Four Forests Restoration Initiative (4FRI). The initiative aims to restore forest densities to presettlement levels in order to reduce wildfire risk and to improve forest health. While provision of additional water supply is not a specific 4FRI target, it is expected to be a side benefit of forest thinning due to the reduction in interception and evapotranspiration losses [30] and to more efficient source-area concentration of snowmelt water [31]. The impacts of forest thinning on water and sediment yields, and the value to society of those impacts, are both open questions. Although catchment experiments have long shown that thinning of coniferous forests increases water yields [32, 33], the net effect on wellbeing once other impacts (e.g., on sediment yields) are taken into account are less clear. In this paper we explore the net effect of forest thinning on water supply to users in the SRP service area, measured in terms of avoided costs of accessing more expensive sources of water supply, such as groundwater and CAP water. In the next section, we present the component models used to project the consequences of land cover change. A third section reports our results, and a final section discusses the significance of these results.

## Theory and Methods

The SRP reservoirs on the Salt and Verde rivers are designed to store and release water for the benefit of downstream users in the Phoenix metropolitan area, smoothing the supply of water relative to natural precipitation and streamflow. The reservoirs also serve as sediment traps, capturing sediment that is transported by the waters. Sediment retention in the watersheds feeding the reservoirs is valuable if sediment delivery impacts reservoir functions in ways that increase costs directly (i.e. through sediment removal) or indirectly (i.e. through a reduction in the reliability of water supplies to downstream water users). If sediment accumulation does not affect reservoir functions, then there is no value to sediment retention for SRP water management. In what follows we assume that the benefits offered by reservoirs are strictly related to their effective storage capacity. That is, we ignore benefits associated with, for example, recreation, local microclimatic effects, impacts on wildlife and so on.

Reservoirs are subject to both within-year (seasonal) and over year (carryover) storage requirements. Within-year systems generally refill each year, whereas over-year systems contain long multiyear drawdown periods and are seldom full [34]. The planning of most surface water reservoirs is based on capacity–yield–reliability methods. Amongst them, the Gould-Dicer method [35] is widely used to estimate capacity–yield–reliability relationships for systems dominated by over-year requirements [36]. This approach is based on the concept of "critical drawdown", that defines the required minimum storage capacity from the historical mean and variability of inflows [36, 37].

We define a watershed production function in which water yields are summed over all spatial units (30m pixels) in the study area—the area of the Salt and Verde watersheds under ponderosa pine (Fig. 1). We consider land cover to be a continuous variable, 0 ≤ *L*_*xj*_ ≤ 1, expressing the percent canopy cover of forest type *j* in pixel *x*. To abstract from changes in water demand in the Phoenix Metropolitan Area, we assume a fixed annual water demand (*Y*) equal to the sum of surface water (*S*) and groundwater supply (*G*). We further assume that surface and groundwater are perfect substitutes in consumption, but involve different marginal costs in acquisition. Both surface and groundwater flows benefit only water users in the SRP "service" area, and the aquifer is assumed not to be physically connected to the upstream watershed by subsurface flows.

Assuming normally distributed and independent annual flows (mean *μ* and standard deviation *σ*), the sum of *t* consecutive annual flows into the reservoir system is given by [36]:

$$X_{q}(t)=t\mu -z\sigma \sqrt{t}$$ (1)

where *z* is the *q* ^*th*^ percentile of a standardized random variable from a normal distribution (*z* < 0 because we are looking at inflows below the mean). The draft over *t* years is defined as:

$$\hat{Y}(t)=tY$$ (2)

where *Y* is the mean annual water demand. Thus, the required storage capacity *V*_*q*_ to meet the target draft *Ŷ* during a *t* year period with reliability (1-*q*) is given by:

$$V_{q}(t)=\hat{Y}(t)-X_{q}(t)$$ (3)

In arid and semi-arid regions, reservoirs are frequently subject to critical periods of drought during which the reservoir contents may decline from full to nearly empty. The length (*h*-years) of the drawdown period is defined in terms of the annual mean and variance of water inflows, the target draft and the probability of non-exceedance (i.e. reliability) by differentiating Eq. (3) with respect to *t* [36]:

$$h={\left(\frac{z\sigma }{2\left(\mu -Y\right)}\right)}^{2}$$ (4)

By substitution of Eq. (4) into Eq. (3) (i.e. *t* = *h*) we obtain the reservoir capacity required to meet water demand during an *h*-year drawdown period with (1-*q*) percent reliability:

$$V_{q}=\frac{z^{2}\sigma^{2}}{4\left(\mu -Y\right)}$$ (5)

Notice that the required reservoir capacity increases with an increase in the variability of annual inflows (standard deviation *σ*), with an increase in the annual target draft, and with a decrease in mean annual inflows (*μ*).

We link the critical drawdown approach [36, 37] to spatially-explicit reservoir sedimentation (see **S1 File**) and hydrological models (see **S2 File**). We then embed the impacts of land cover change on reservoir storage into an optimal land management problem. Given the initial capacity of the reservoir system *V*_*0*_, and after adjusting for mean annual net water evaporation (i.e. evaporation net of direct precipitation) from the reservoir surface (Table 1), *V*_*t*_ < *V*_*0*_ is the reduced active storage capacity of the reservoir after *t* years of cumulative sedimentation.

Land cover change impacts mean annual inflow through its effects on annual evapotranspiration, which takes water out of the system. Assuming that *V*_*t*_ = *V*_*q*_ at any time *t*, from Eq. (5) we can express the change in the annual probability (1−*q*_*t*_(*z*_*t*_)) for delivering the amount of water demand *Y*, as function of both land cover change in the basin and sediment accumulation over time:

$$z_{t}\left(L_{xi}\right)=\frac{2}{\sigma }\sqrt{V_{t}(L_{xi})\left(\mu (L_{xi})-Y\right)}$$ (6)

The annual standard deviation of water inflows, *σ*, is assumed to vary proportionally (defined by observed standard deviation vs. observed mean inflow at the baseline landscape) with changes in the inflows due land cover change. Thus, the expected surface water supply (*S*_*t*_) is identified by the annual reliability 0 < *q*_*t*_(*z*_*t*_) < 0.5 for delivering an amount equivalent to the water demand *Y*:

$$S_{t}(L_{xi})=\left(1-q_{t}(z_{t})\right)Y$$ (7)

From Eq. (6), if water demand is close to mean annual inflow (*Y* ≃ *μ*(*L*_*xi*_)) then surface water supply satisfies only about half of the required target (i.e. *q*_*t*_(*z*_*t*_) ≃ 0.5). Complementary sources, such as groundwater uptake in the SRP service area (*G*_*t*_) and CAP water (*C*_*t*_), make up the balance. Thus, the total water demand-supply balance is defined by:

$$Y=S_{t}+G_{t}+C_{t}=Y\left(1-q_{t}(z_{t})\right)+G_{t}+C_{t}$$ (8)

**Fig 2:**
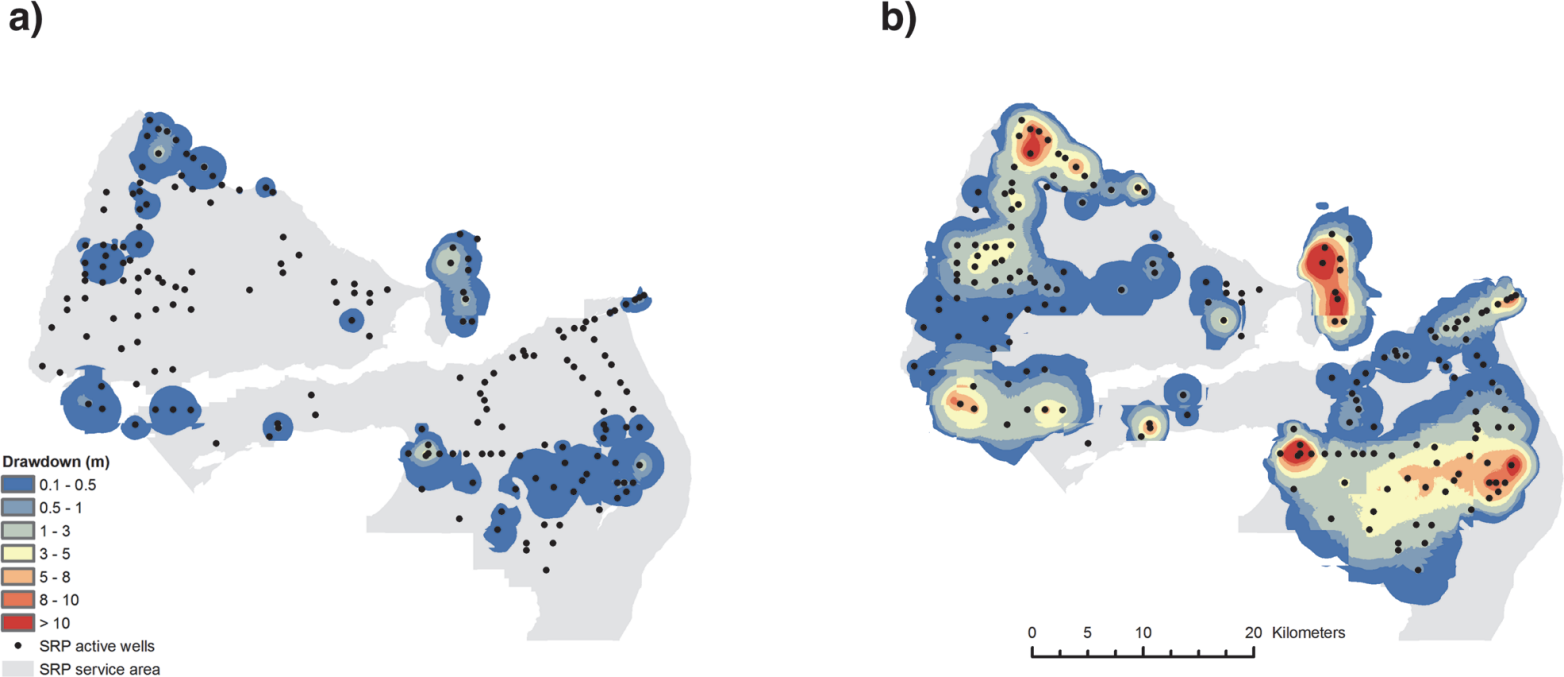
Estimated drawdown effect of groundwater pumping from SRP wells. a) baseline uptake at year 0; b) maximum groundwater uptake of 325,000 AF year^-1^.

The target for alternative water supplies is the difference between annual water demand and the expected surface water supply, and is identified by the annual probability of surface water delivery failure, *G*_*t*_ + *C*_*t*_ = *Yq*_*t*_(*z*_*t*_). The share of groundwater and CAP water supply depends on the ratio of their respective supply costs. Both SRP surface water price (*p*_*s*_) and CAP water price (*p*_*c*_) are assumed to be constant and identified by the values reported in 2013, *p*_*s*_ = 14$ AF^-1^ and *p*_*c*_ = 120 $ AF^-1^. Groundwater prices (*p*_*gt*_), on the other hand, are estimated in terms of variable pumping costs. For the *n* wells in the SRP service area, the pumping cost is related to the depth of the water table at each well:

$$p_{gt}=\frac{\sum_{j=1}^{n}p_{kwh}E_{jt}Q_{jt}}{n}$$ (9)

with

$$\sum_{j=1}^{n}Q_{jt}=G_{t}(L_{xi})$$ (10)

$$E_{jt}=\frac{0.736}{270}\frac{U_{jt}}{\eta_{j}}$$ (11)

$$U_{jt}=H_{j}+\sum_{j}h_{jt}(Q_{jt})$$ (12)

where *Q*_*jt*_ represents annual groundwater uptake (m^3^) from the *j* ^*th*^ well; *E*_*jt*_ is the energy in Kwh/m^3^ required at each *j* ^*th*^ well to lift one cubic meter of water to a vertical distance *U*_*jt*_ expressed in meters; *p*_*kwh*_ is the unit price of energy assumed at 0.10 $; and 0 ≤ *η*_*j*_ ≤ 1 is the pumping efficiency of the well assumed as 0.90 for all the wells; *H*_*j*_ represents the vertical distance of the water table at the steady state and is taken from a 2006 water table map [38]; and *h*_*jt*_ is the aquifer drawdown at time *t* which is a function of the intersection of the drawdown from the *j* ^*th*^ well and multiple cones of depression generated by water pumping from neighbouring wells (Fig. 2). Aquifer drawdown at the j*th* well increases with increasing pumping rate *Q*_*jt*_ and it returns to zero when pumping stops. At any time *t*, pumping rates at each well are estimated from the gap between effective surface water delivery and target supply (*Y* − *S*_*t*_ = *G*_*t*_ = ∑_*j*_ *Q*_*jt*_), assuming uniform percentage variation (*α*) for all wells (i.e. ∑_*j*_ *αQ*_*jt*_ = *αG*_*t*_) from the 2006 groundwater pumping data provided for 183 SRP active wells (Fig. 2) within the Phoenix metropolitan area [38]. In doing this we ignore any potential limit to pumping capacity at each well. Eq. (9) says that the price of groundwater is affected by variations in the reliability of surface water supply, thus it is also a function of land cover change in the upstream basin. The aquifer drawdown model is described in **S3 File**.

Assuming *p*_*gt*_ < *p*_*c*_ at any *t*, groundwater may be used as a substitute for surface water up to the maximum sustainable pumping (*Ĝ*) rate, which for the sum of all SRP wells is estimated to be 325,000 AF year^-1^. Above this threshold CAP water replaces surface water supply for the additional amount required:

$$\left\{ \begin{array}{l} S_{t}(L_{xi})=\left(1-q_{t}(z_{t})\right)Y\\ G_{t}(L_{xi})=Yq_{t}(z_{t}),\;Y-S_{t}\leq \hat{G}\\ C_{t}(L_{xi})=Yq_{t}(z_{t})-\hat{G},\;Y-S_{t}>\hat{G} \end{array}\right.$$ (13)

The annual probability of surface water delivery failure gives the expected amount of alternative water supplies, and is affected by changes in both water yields and sediment accumulation in the reservoirs *D*(*L*_*xi*_). This is taken to be a function of land cover in the watershed. So reservoir capacity evolves according to:

$$V_{t}\left(L_{xi}\right)=V_{0}-tD\left(L_{xi}\right)$$ (14)

where *V*_*0*_ is the initial storage capacity of the system: 2.853 million m^3^, reduced to 2.765 million m^3^ after accounting for evaporation and direct precipitation (Table 1). *V*_*t*_ declines from this level as a function of the annual sedimentation rate *D*, and time.

It follows that forest thinning may reduce the cost of surface water supply by increasing surface water reliability, which leads to a reduction in demand for more costly alternatives such as groundwater. However, from Eq. (6) the aggregate impact of canopy cover reduction across the basin can be ambiguous since the effect of increased mean annual inflow and the decreased reservoir storage capacity from sedimentation have opposite effects on surface water supply reliability.

Now consider what this implies for the cost of water. The net present value of the spatially distributed effect of forest management interventions such as canopy reduction (*L*_*xi*_) is measured in terms of mean annual avoided cost, identified by the marginal variation of the standardized random variable (*z*_*t*_) from the normal distribution of the probability function (*q* = *f*(*z*_*t*_)). Despite constant water demand, this marginal change varies with *t* due to sediment accumulation:

$$\frac{\partial z_{t}}{\partial L_{xi}}=\frac{-t\left(\frac{\partial D}{\partial L_{xi}}\right)\left(\mu -Y\right)+\left(V_{0}-tD\right)\left(\frac{\partial \mu }{\partial L_{xi}}\right)}{\sigma \sqrt{\left(V_{0}-tD\right)\left(\mu -Y\right)}}$$ (15)

It follows that the marginal change in the share of total water supply from each source depends on the marginal change in the expected probability of surface water delivery failure:

$$\left\{ \begin{array}{l} \frac{\partial S_{t}}{\partial L_{xi}}=\beta_{t}\left[q\left(z_{t}\right)-q\left(z_{t}+\frac{\partial z_{t}}{\partial L_{xi}}\right)\right]Y\\ \frac{\partial G_{t}}{\partial L_{xi}}=Y-\frac{\partial S_{t}}{\partial L_{xi}},\;Y-S_{t}\leq \hat{G}\\ \frac{\partial C_{t}}{\partial L_{xi}}=Y-\frac{\partial S_{t}}{\partial L_{xi}},\;Y-S_{t}>\hat{G} \end{array}\right.$$ (16)

where *β*_*t*_ is a basin-specific scaling factor representing the ratio between the sum of the marginal variations in surface water supply from all the pixels and the variation in surface water supply for the entire basin. This adjustment is required since the marginal variation at each pixel cannot be estimated simultaneously with the marginal variation from all the other pixels. Thus, over a *T*-year planning horizon and a given discount rate *r*, the net present value of the forest management regime (i.e. thinning) for the *i* ^*th*^ forest type at the *x* ^*th*^ pixel is:

$$NPV_{L_{xi}}=\sum_{t=1}^{T}\left(p_{s}\frac{\partial S_{t}}{\partial L_{xi}}+p_{gt}\frac{\partial G_{t}}{\partial L_{xi}}+p_{c}\frac{\partial C_{t}}{\partial L_{xi}}\right){\left(1+r\right)}^{-t}$$ (17)

If we assume that water demand is not fixed but it grows with time at a constant rate, the share of total water supply from each source is given by:

$$\left\{ \begin{array}{l} z_{0t}\left(L_{xi}\right)=\frac{2}{\sigma }\sqrt{V_{t}(L_{xi})\left(\mu (L_{xi})-Y_{0}\right)}\\ z_{t}\left(L_{xi}\right)=\frac{2}{\sigma }\sqrt{V_{t}(L_{xi})\left(\mu (L_{xi})-Y_{t}\right)}\\ S_{t}(L_{xi})=\left(1-q_{0t}(z_{0t})\right)Y_{0}+\sum_{t=1}\left(1-q_{t}(z_{t})\right)\left(Y_{t}-Y_{t-1}\right)\\ G_{t}(L_{xi})=Y_{0}q_{0t}(z_{0t})+\sum_{t=1}\left(Y_{t}-Y_{t-1}\right)q_{t}(z_{t}),\;Y_{t}-S_{t}\leq \hat{G}\\ C_{t}(L_{xi})=Y_{0}q_{0t}(z_{0t})+\sum_{t=1}\left(Y_{t}-Y_{t-1}\right)q_{t}(z_{t})-\hat{G}\;,\;Y_{t}-S_{t}>\hat{G} \end{array}\right.$$ (18)

and the marginal variations in each share is:

$$\left\{ \begin{array}{l} \frac{\partial S_{t}}{\partial L_{xi}}=\beta_{t}\left[\left(q\left(z_{0t}\right)-q\left(z_{0t}+\frac{\partial z_{0t}}{\partial L_{xi}}\right)\right)Y_{0}+\sum_{t=1}\left(q\left(z_{t}\right)-q\left(z_{t}+\frac{\partial z_{t}}{\partial L_{xi}}\right)\right)\left(Y_{t}-Y_{t-1}\right)\right]\\ \frac{\partial G_{t}}{\partial L_{xi}}=Y_{t}-\frac{\partial S_{t}}{\partial L_{xi}},\;Y_{t}-S_{t}\leq \hat{G}\\ \frac{\partial C_{t}}{\partial L_{xi}}=Y_{t}-\frac{\partial S_{t}}{\partial L_{xi}},\;Y_{t}-S_{t}>\hat{G} \end{array}\right.$$ (19)

At time *t* = *n*, when the total water demand becomes higher than the mean annual inflow to the reservoirs system with *Y*_*n*_ ≥ *μ* and *Y*_*n*−1_ < *μ*, the expected surface water supply cannot be increased, thus reaching its maximum(*Ŝ*_*t*_). This maximum amount however decreases with time due to sediment accumulation reflected by the increasing trend in *q*_0*t*_(*z*_0*t*_):

$$\left\{ \begin{array}{l} z_{0t}\left(L_{xi}\right)=\frac{2}{\sigma }\sqrt{V_{t}(L_{xi})\left(\mu (L_{xi})-Y_{0}\right)}\\ q_{n}=0.5\\ {\hat{S}}_{t}(L_{xi})=\left(1-q_{0t}(z_{0t})\right)Y_{0}+\sum_{t=1}^{n-1}\left(1-q_{t}(z_{t})\right)\left(Y_{t}-Y_{t-1}\right)+\left(\mu -Y_{n-1}\right)q_{n}\\ G_{t}=Y_{t}-{\hat{S}}_{t}\;,\;Y_{t}-{\hat{S}}_{t}\leq \hat{G}\\ C_{t}=Y_{t}-{\hat{S}}_{t}-\hat{G}\;,\;Y_{t}-{\hat{S}}_{t}>\hat{G} \end{array}\right.$$ (20)

Using this approach we estimated the spatial distribution of the marginal impact of a proportional canopy cover reduction, measured in terms of the water delivery costs required to meet a given annual water supply. The spatial analysis was conducted at a 30m by 30m pixel resolution. The land use and land cover baseline was represented (a) by a reclassified land cover map, based on elevation and precipitation ranges, obtained from the National Land Cover Dataset (NLCD) for year 2001, and (b) by the NLCD 2001 percent canopy cover map applied to forest cover vegetation. The hydrological model was calibrated on mean annual precipitation and hydrograph data for the period 1995–2005. The sediment delivery model was calibrated against observed data from the 1995 sedimentation survey of Roosevelt Lake, which reported a mean annual storage capacity loss of 2.62 million m^3^ (2,121 AF) over a 85-year period [39]. We calibrated the model using precipitation data for the period 1970–2000. Land use and land cover parameters were obtained from the 2001 maps used to calibrate the hydrological model.

The water yield model was calibrated and validated on data from three USGS hydrological stations, representing three sub-basins—the Verde River basin above Horseshoe Lake, the Salt River basin above Roosevelt Lake, and the Tonto river basin above Roosevelt Lake—for the period 1995–2005. Yields were adjusted based on observed anthropogenic water consumption in each of the three sub-basins [40]: 57.85, 30.47 and 5.80 million m^3^ year^-1^ for Verde, Salt and Tonto River basin respectively. After calibration, estimated mean annual water yields ranged from 10% overestimation for the Verde sub-basin to -7% and -18% underestimation for the Salt and Tonto sub-basins respectively. The flow from the Tonto sub-basin represents only about 7% of the annual water yield from the entire SRP basin, thus diminishing the relative weight of the high underestimation of predicted water yield from this watershed. For the entire SRP basin the estimated annual water yield, net of upstream water consumption, was within 0.7% of observed flows The estimated mean annual storage capacity loss was 2.59 million m^3^ (2,096 AF), within 1.2% of the observed rate from the 1995 survey of Roosevelt Lake. Since there are no comparable sediment surveys for the Horseshoe reservoir on the Verde River, calibration for the entire SRP basin was based on the Salt River sub-basin only.

## Results

According to SRP Daily Water Reports, mean annual SRP water supply during the period 2008–2011 was around 1,024 million m^3^, 93% of which was accounted for by surface water and 7% by groundwater. Our model estimates applied to the baseline land cover match those numbers. We estimated mean annual inflows of about 1,187 million m^3^ into the SRP reservoir system, which translates in an expected mean annual surface water supply of about 980 million m^3^. Consequently, under the assumption that the reported mean annual SRP supply for the period 2008–2011 identifies the current annual water demand at 1,024 million m^3^ (830,000 AF), the SRP groundwater supply required for meeting the annual target delivery is about 44 million m^3^.

**Fig 3:**
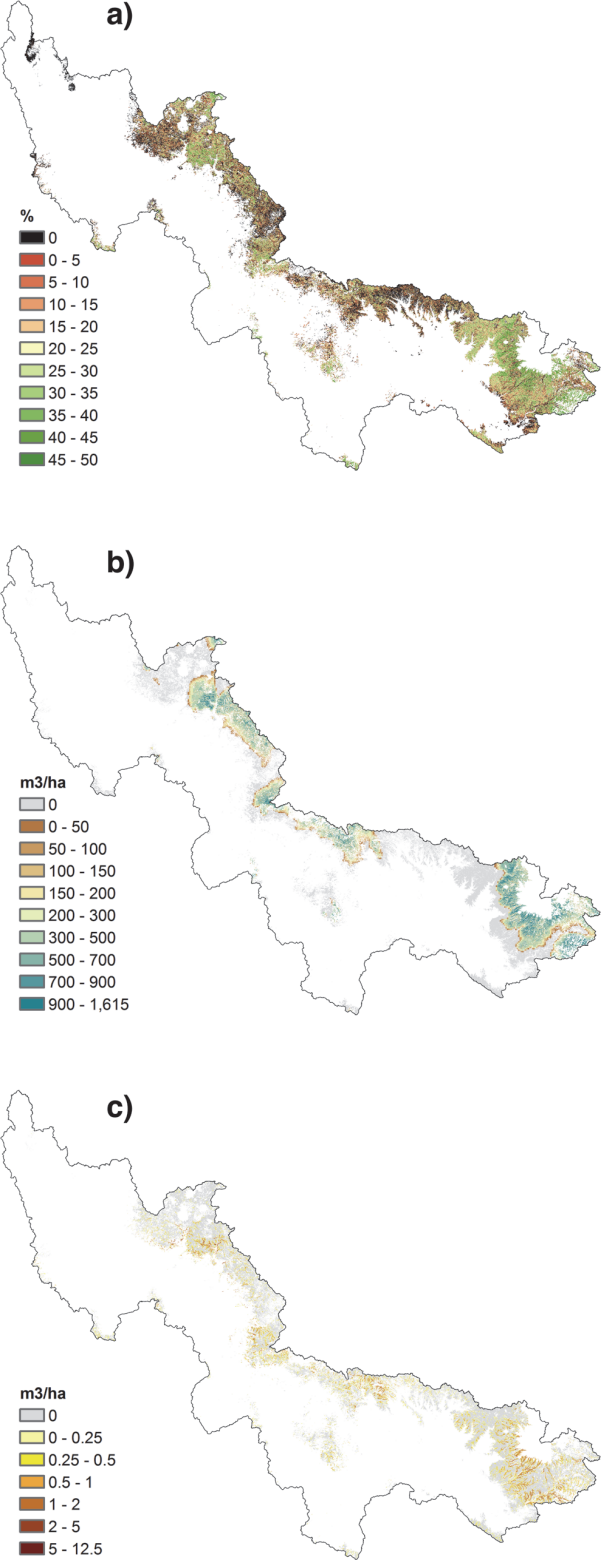
Estimated impact of forest management in the SRP basin. a) Simulated percent canopy cover reduction under forest restoration for ponderosa pine forest; b) Estimated annual water yield (m^3^) variation from forest thinning; c) Estimated marginal impact of forest thinning in terms of annual storage capacity loss (m^3^) to the SRP reservoir system.

To estimate the impact of a change in land cover on water and sediment yields, and water costs, we considered the effect of an arbitrary change in canopy cover in the ponderosa pine forest that occupies the upper part of Salt and Verde watersheds. The 4FRI project envisages a 50% reduction in basal area in the ponderosa pine forest. We similarly restricted ourselves to ponderosa pine forest, but instead of a 50% reduction in basal area, we considered a reduction to 50% canopy cover. Our aim was not to investigate the implications of 4FRI in particular, but to evaluate the effect of variable rates of thinning on the cost of water delivery. Structuring the thinning experiment in this way made it possible to spatially differentiate the potential contributions of different parts of the watershed. Since current canopy cover for ponderosa pine forest is higher in the Salt River basin relative to Verde River watershed, this implied a greater reduction in standing biomass in the Salt than in the Verde (Fig. 3A). All land cover other than ponderosa pine (piñon-juniper, subalpine forest, evergreen-deciduous mixed forest) and all ponderosa pine forest where current canopy cover is already at or below 50% were left unchanged. We also assume that canopy cover reduction takes place at the same time for all the pixels and it is maintained over time. We do not account for the management cost of maintaining lower canopy cover after thinning, assuming it will be naturally maintained by fire over a 20-year cycle. While the experiment is arbitrary, it helps us to illustrate the water-sediment trade-offs involved in different forest conditions.

Using 1995–2005 mean annual precipitation data as input in our model, we found that canopy reduction to 50% in the ponderosa pine forest would increase annual water yield in the SRP watershed by 8.2% (Fig. 3B and Table 2). We found that annual sedimentation rates would also increase, by 1.5% (Fig. 3C and Table 2), but sediment loads building up over time had a negligible average impact on the annual probability of surface water delivery failure, even though there was some degree of spatial variability across the watershed. We take the difference in SRP revenues from water sales with and without thinning as a first approximation of the water value of thinning, bearing in mind that the price of water is significantly below its social opportunity cost.

**Table 2:** Estimated mean annual water yield and storage capacity loss under baseline 2001 LULC and 50% canopy cover scenario for Ponderosa pine forest.

Basin	Water yield				Storage capacity loss			
	Baseline		Canopy reduction		Baseline		Canopy reduction	
	AF year^-1^	million m^3^ year^-1^	AF year^-1^	million m^3^ year^-1^	AF year^-1^	million m^3^ year^-1^	AF year^-1^	million m^3^ year^-1^
Salt & Tonto River	549,791	678.16	602,803	743.55	2,096	2.59	2,139	2.64
Verde River	412,603	508.94	438,933	541.42	1,971	2.43	1,991	2.46
Total SRP	962,394	1,187.10	1,041,736	1,284.96	4,067	5.02	4,130	5.10

**Fig 4:**
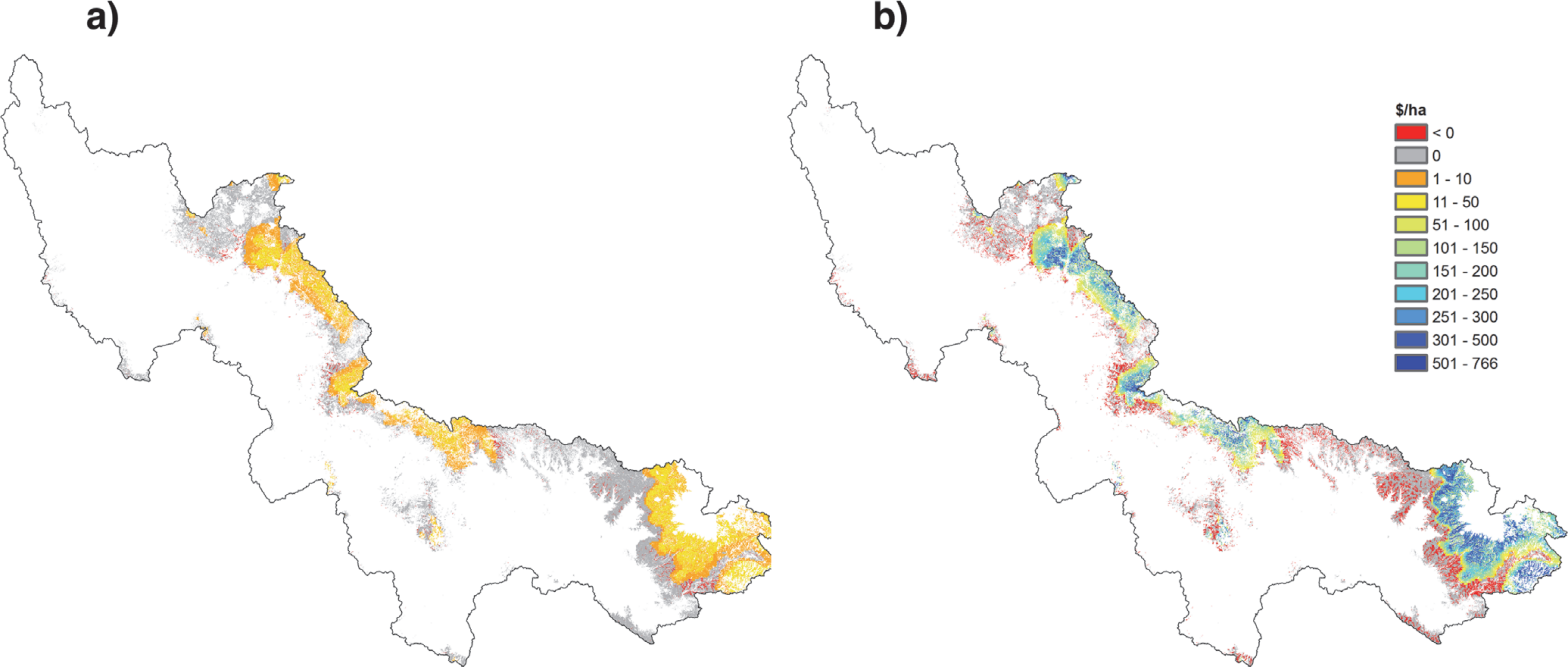
Net Present Value (NPV) of forest thinning over 20 years across the SRP watershed. a) constant water demand; b) 2.7% annual increase in water demand.

Taken over a 20-year time horizon, the net present value (NPV) of the reduced cost of water from avoided groundwater and CAP water use, was found to be US$ 2.4 million at a 2% discount rate (Fig. 4A and Table 3). Assuming constant water demand, thinning at this level does result in a slight increase in expected surface water supply, and a matching reduction in groundwater pumping. The reduction in pumping generates a lower drawdown effect at the wells, leading to a fall in average pumping cost. This produces a small reduction in the mean water price over 20 years from 14.4 to 14.2 $ AF^-1^.

**Table 3:** Predicted water supply scenarios from forest management in the SRP watershed over a 20-year period with constant water demand.

Year	Total water supply (AF)***Y***_*t*_	Avoided water supply cost from forest management(UNDISCOUNTED)	NPV of avoided water supply cost from forest management(DISCOUNTED)	Mean water price($ AF^-1^)		Mean Groundwaterpumping cost ($ AF^-1^)		Surface water% of total supply***S***_*t*_		Groundwater % of total supply***G***_*t*_		CAP water % of total supply***C***_*t*_	
				Baseline	Canopy reduction	Baseline	Canopy reduction	Baseline	Canopy reduction	Baseline	Canopy reduction	Baseline	Canopy reduction
0	830,000	142,873	142,873	14.36	14.18	19.84	19.20	93.9	96.5	6.1	3.5	0.0	0.0
1	830,000	143,253	140,444	14.36	14.18	19.84	19.20	93.9	96.5	6.1	3.5	0.0	0.0
2	830,000	143,634	138,057	14.36	14.18	19.85	19.21	93.9	96.5	6.1	3.5	0.0	0.0
3	830,000	144,016	135,710	14.36	14.19	19.85	19.21	93.9	96.4	6.1	3.6	0.0	0.0
4	830,000	144,399	133,403	14.36	14.19	19.86	19.21	93.8	96.4	6.2	3.6	0.0	0.0
5	830,000	144,784	131,135	14.36	14.19	19.86	19.22	93.8	96.4	6.2	3.6	0.0	0.0
6	830,000	145,169	128,906	14.36	14.19	19.86	19.22	93.8	96.4	6.2	3.6	0.0	0.0
7	830,000	145,555	126,715	14.36	14.19	19.87	19.22	93.8	96.4	6.2	3.6	0.0	0.0
8	830,000	145,943	124,561	14.37	14.19	19.87	19.23	93.8	96.4	6.2	3.6	0.0	0.0
9	830,000	146,332	122,444	14.37	14.19	19.88	19.23	93.8	96.4	6.2	3.6	0.0	0.0
10	830,000	146,722	120,363	14.37	14.19	19.88	19.23	93.7	96.3	6.3	3.7	0.0	0.0
11	830,000	147,113	118,317	14.37	14.19	19.89	19.24	93.7	96.3	6.3	3.7	0.0	0.0
12	830,000	147,505	116,306	14.37	14.19	19.89	19.24	93.7	96.3	6.3	3.7	0.0	0.0
13	830,000	147,898	114,330	14.37	14.19	19.89	19.24	93.7	96.3	6.3	3.7	0.0	0.0
14	830,000	148,292	112,387	14.37	14.19	19.90	19.25	93.7	96.3	6.3	3.7	0.0	0.0
15	830,000	148,688	110,477	14.37	14.20	19.90	19.25	93.7	96.3	6.3	3.7	0.0	0.0
16	830,000	149,085	108,600	14.38	14.20	19.91	19.25	93.6	96.3	6.4	3.7	0.0	0.0
17	830,000	149,482	106,755	14.38	14.20	19.91	19.26	93.6	96.3	6.4	3.7	0.0	0.0
18	830,000	149,881	104,941	14.38	14.20	19.92	19.26	93.6	96.2	6.4	3.8	0.0	0.0
19	830,000	150,282	103,158	14.38	14.20	19.92	19.26	93.6	96.2	6.4	3.8	0.0	0.0
20	830,000	150,683	101,405	14.38	14.20	19.93	19.27	93.6	96.2	6.4	3.8	0.0	0.0
		2,938,715	2,398,413	14.37	14.19								

The marginal water value of forest thinning is, however, sensitive to the assumption made about the level of aggregate water supply. It is also sensitive to the marginal price of surface water, and the cost of pumping. In a second experiment we projected an increase in water demand equivalent to population growth projections (2.7% per year) (Table 4). Once again this is arbitrary. There is no special reason to believe that water demand would increase proportionately. Our aim was to test the sensitivity of the revenue impact of thinning to growth in demand. Under this scenario, the NPV of avoided water supply cost from forest thinning was estimated to be US$ 45.4 million (Fig. 4B). With current canopy cover, increasing water demand would require increasing groundwater supply, reaching the maximum SRP pumping capacity of 401 million m^3^ at year 15, with an average pumping cost of 28.07 $ AF^-1^. At this point more expensive CAP water would be alternatively supplied at increasing rates. Under forest thinning, however, the maximum groundwater supply would be reached at year 18 with a lower mean water price over 20 years of 16.1 $ AF^-1^ compared to 18.4 $ AF^-1^ without forest thinning.

**Table 4:** Predicted water supply scenarios from forest management in the SRP watershed over a 20-year period with 2.7% annual water demand increase.

Year	Total water supply (AF)***Y***_*t*_	Avoided water supply cost from forest management(UNDISCOUNTED)	NPV of avoided water supply cost from forest management(DISCOUNTED)	Mean water price($ AF^-1^)		Mean Groundwaterpumping cost ($ AF^-1^)		Surface water% of total supply***S***_*t*_		Groundwater % of total supply***G***_*t*_		CAP water % of total supply***C***_*t*_	
				Baseline	Canopy reduction	Baseline	Canopy reduction	Baseline	Canopy reduction	Baseline	Canopy reduction	Baseline	Canopy reduction
0	830,000	142,873	142,873	14.36	14.18	19.84	19.20	93.9	96.5	6.1	3.5	0.0	0.0
1	852,410	150,454	147,504	14.36	14.19	19.90	19.23	93.8	96.4	6.2	3.6	0.0	0.0
2	875,425	161,350	155,085	14.38	14.19	19.97	19.27	93.7	96.4	6.3	3.6	0.0	0.0
3	899,062	177,604	167,360	14.40	14.20	20.08	19.33	93.5	96.3	6.5	3.7	0.0	0.0
4	923,336	203,271	187,791	14.43	14.21	20.23	19.40	93.1	96.1	6.9	3.9	0.0	0.0
5	948,266	249,034	225,558	14.49	14.22	20.47	19.49	92.5	95.9	7.5	4.1	0.0	0.0
6	973,869	338,072	300,198	14.59	14.25	20.85	19.61	91.3	95.6	8.7	4.4	0.0	0.0
7	1,000,164	566,637	493,291	14.85	14.28	21.65	19.78	88.9	95.1	11.1	4.9	0.0	0.0
8	1,027,168	820,950	700,673	15.14	14.34	22.46	20.04	86.6	94.4	13.4	5.6	0.0	0.0
9	1,054,902	1,080,844	904,402	15.46	14.44	23.30	20.46	84.3	93.2	15.7	6.8	0.0	0.0
10	1,083,384	1,243,492	1,020,097	15.82	14.68	24.16	21.32	82.1	90.8	17.9	9.2	0.0	0.0
11	1,112,636	1,410,620	1,134,509	16.22	14.95	25.04	22.20	79.9	88.4	20.1	11.6	0.0	0.0
12	1,142,677	1,582,349	1,247,672	16.66	15.27	25.94	23.11	77.8	86.0	22.2	14.0	0.0	0.0
13	1,173,529	1,758,806	1,359,614	17.13	15.63	26.87	24.04	75.7	83.8	24.3	16.2	0.0	0.0
14	1,205,214	1,940,119	1,470,368	17.64	16.03	27.83	24.99	73.7	81.6	26.3	18.4	0.0	0.0
15	1,237,755	4,125,079	3,064,995	19.80	16.46	28.07	25.97	71.8	79.4	26.3	20.6	2.0	0.0
16	1,271,175	6,991,653	5,093,041	22.44	16.94	28.07	26.98	69.9	77.3	25.6	22.7	4.6	0.0
17	1,305,496	9,865,087	7,045,276	25.02	17.46	28.07	28.01	68.0	75.3	24.9	24.7	7.1	0.0
18	1,340,745	10,048,094	7,035,268	27.53	20.03	28.07	28.07	66.2	73.3	24.2	24.2	9.5	2.5
19	1,376,945	10,051,598	6,899,726	29.97	22.67	28.07	28.07	64.5	71.3	23.6	23.6	11.9	5.0
20	1,414,122	10,055,101	6,766,795	32.35	25.24	28.07	28.07	62.8	69.5	23.0	23.0	14.3	7.6
		62,820,214	45,419,220	18.43	16.09								

**Fig 5:**
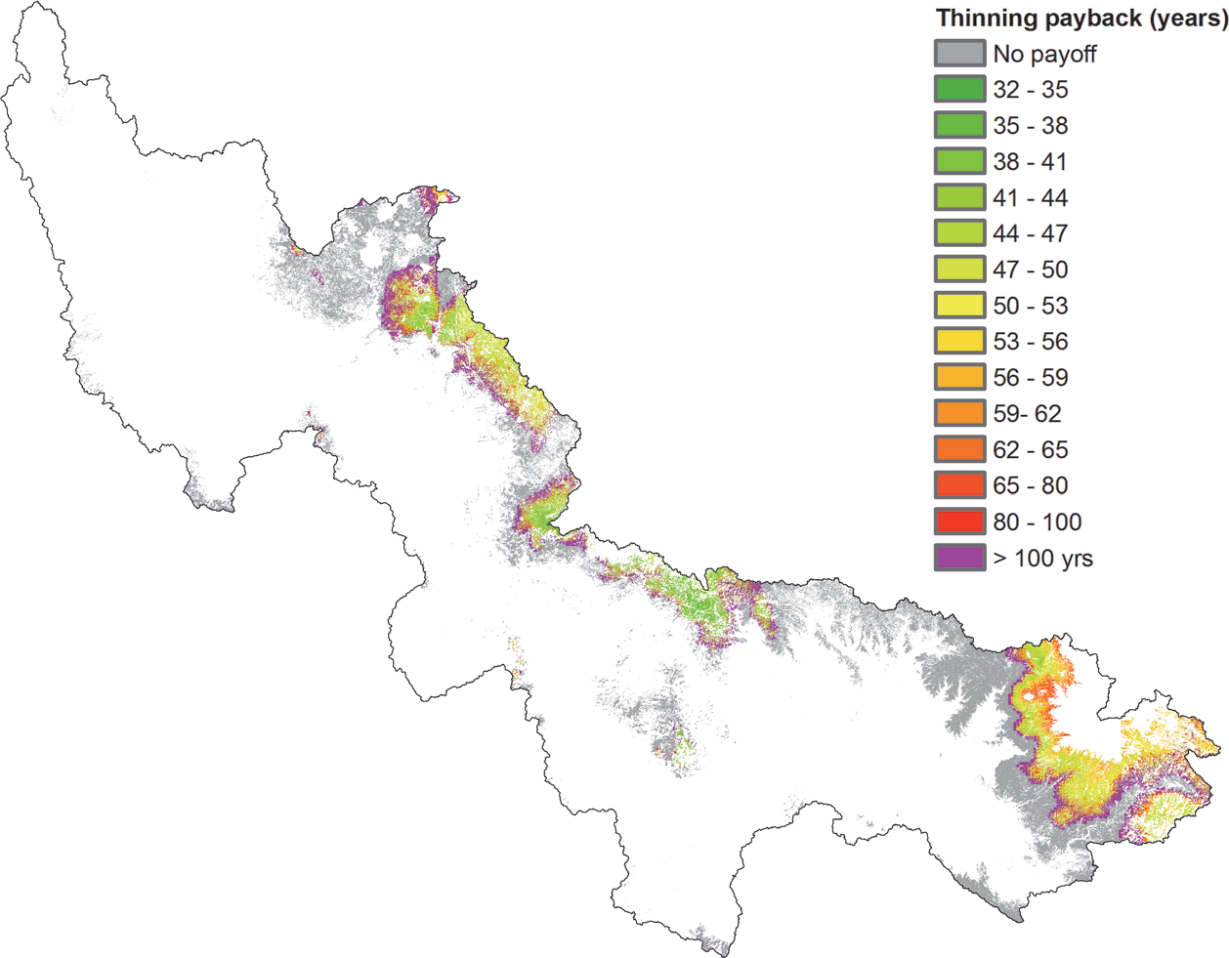
Payback years of forest thinning.

Both scenarios assume that the impacts on water and sediment are an incidental or external effect of forest thinning that is occurring for other reasons, such as fire regulation. To see whether thinning might be justified in terms of water and sediment impacts alone we should set the net benefits of thinning against the cost of thinning. Operational costs of thinning can be high and spatially variable. It is estimated that harvest costs for restoration treatments in the White Mountains of the Apache-Sitgreaves National Forests could range between $1,100 and $1,300 per acre, while in the Coconino and Kaibab National Forests harvest costs may range between $557 and $836 per acre [41]. Considering the latter value represents canopy reduction from 100% to 50% and assuming a linear relationship in the cost of thinning, this would be 41.32 $ ha^-1^ for 1% canopy cover reduction. Applying this cost to the thinning scenario (Fig. 3A), and assuming 2.7% annual water demand growth, we can map the spatial distribution of the payback time of forest management across the SRP watershed (Fig. 5). This varies between 32 and 100 years or more. In other words, the water-related benefits from forest thinning would offset operational costs only for time horizons of 32 years or more. However, water yield response to overstory reduction treatment will generally be lost after 6–10 years, depending on sun exposure related to aspect [31]. Since we ignore natural regrowth, assuming that the thinned forest is thereafter regulated through natural fire, the number of years needed to pay back investment in thinning could be even higher than shown. On the other hand, if the value of fire regulation or water quality affected by large fire events would be added into the analysis, the payback time would be much shorter. Not to mention that thinned forests of ponderosa pine in the southwestern United States are a desirable alternative to intensively burned forests to maintain carbon stocks and primary production, as well as in reducing the limitation of drought on carbon uptake during summer [42].

**Fig 6:**
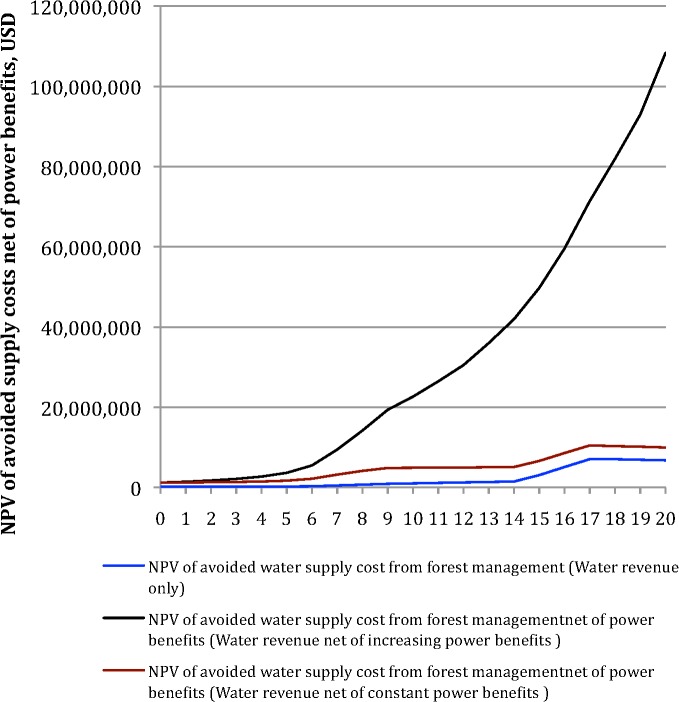
Net present value of the water-related benefits of forest thinning under different assumptions about the benefits of surface water to the generation of electric power.

Because the price paid by consumers is below the true social opportunity cost of water we also considered the sensitivity of this result to the size of (at least) direct subsidies. We take the contributions to support water operations from electricity revenues within SRP to be a first approximation of the value of surface water storage for power generation. If the 2013 subsidy is used to project future values, we find that the NPV of the water-related benefits of thinning rises from $45.4 million to $103.7 million. Moreover, if the subsidy were to continue to rise at the same rate as it has from 2010, the NPV of the water-related benefits of thinning rises to $683 million (Fig. 6). There is no reason to believe that the subsidy will continue to rise at the same rate for the next 20 years, but the scenario illustrates how sensitive the water value of thinning is to assumptions made about water benefits beyond direct consumption.

## Discussion

Any impact of forest thinning on water supply in Central Arizona is considered by the USDA to be an incidental benefit of management. Nevertheless, it is potentially important. While population growth and the expansion of the regional economy are expected to increase future water demand over present levels, climate change is expected to reduce precipitation in the three watersheds responsible for meeting the bulk of water needs: the Colorado, Salt and Verde. Changes in land use and land cover in these watersheds have the potential to alter both water and sediment yields, with opposite effects on the reliability of water supply.

The 4FRI conjecture—that forest thinning would have positive impacts on water supply—is founded on the fact that in almost every catchment-level study, thinning has increased water yields. However, thinning is also expected to influence sediment flows. Since the SRP system relies on over-year reservoirs to maintain supply in conditions of low and highly variable rainfall, we modeled the net effect of thinning on the reliability of water supply, taking both water yield and reservoir sedimentation into account. While we did not model the effects of individual rainfall events, and therefore could not capture the sediment impact of any future change in the frequency of extreme rainfall events, we conclude that the sediment impacts of forest thinning of up to 50% of canopy cover is unlikely to compromise the reliability of the reservoir system. We are more confident about the impact of thinning on reservoirs in the Salt River basin than in the Verde River Basin. The lack of sediment surveys in the latter makes it impossible to calibrate the sediment delivery models on Verde data. Nevertheless, the model errors would have to be very large to reverse the conclusion that thinning enhances net water supply.

The outcome of our analysis is also affected by climate and atmospheric CO2 fluctuations. Water use efficiency, for instance, increases with increasing concentrations of carbon dioxide [43], due to reduced leaf conductance of water [44]. Considering expected climate change scenarios, this may have a strong effect on forests, as plants would be able to function better under drier conditions, even though improvements in water use efficiency may not always offset the effects of reduced precipitation and/or increased temperatures [45]. However, it is not clear if our estimates of forest thinning in terms of increased annual water yield would be negatively affected, since during droughts the importance of increasing direct run-off and decreasing canopy interception, both associated with thinning, may be relatively higher.

Beyond the physical effects of forest thinning on the reliability of water supplies, we were also interested in the value of the enhanced water yields associated with thinning. As noted above, our estimates of the net present value of water if approximated by water revenues to SRP are very small, even if we consider increases in demand above historic rates. This is largely because the price paid by consumers does not reflect the true social opportunity cost of water. Indeed, it does not even reflect the marginal cost of supply via the hard infrastructure. In 2013 SRP had total operating revenues of $2,824 million, but water revenues of only $15 million. Over 90% of operating revenue derives from retail power sales. The SRP power system is not, however, independent of the water supply system. It includes five hydroelectric plants generating 238 megawatts. While this is a relatively small proportion of total SRP capacity (8000 megawatts), it adds important flexibility to the system. For example, power from pumped storage units at Horse Mesa and Mormon Flat Dams can be reversed in off-peak periods to pump water from the lower reservoir back to the upper reservoir for repeated usage. The water price paid by wholesale customers of SRP benefits from a cross subsidy from the utility’s power operations that has been rising sharply in recent years. In 2010, electric revenue contributions to support water operations were 175% of water revenues. In 2013, they stood at 359% of water revenues.

Our results are also sensitive to the assumptions made about the elasticity of water demand. In [46] we estimated the short and long run price and income elasticities of water demand in the SRP supply area of Phoenix and found the price elasticity of demand to be increasing over time. We also found water use to be increasing in income, with low water users (lower income users) more sensitive to increasing prices than high water users (higher income users). Assuming that income growth continues at the average rate of the last decade, we found the NPV of the water benefits of forest thinning to be reduced relative to the base case. It is worth repeating, though, that since water prices are heavily subsidized, the impact of price-induced demand reductions is small.

In Arizona, as elsewhere in the Southwest, water prices do not respond in the short term to changes in water supply. In these circumstances our first approximation of the water value of forest thinning, the change in the cost of supply to municipalities, is likely to be an underestimate. The cross subsidy between power and water insulates consumers from the direct impact of changing water yields. While we are unable to assign all of the electric revenue contributions to support water operations to changing costs of supply, it is clear that one of the reasons for the increase in the size of the subsidy over the last five years is an increase in the cost of water operations.

Of the two main elements in an integrated model of water supplies, there may be least uncertainty about the impact of land cover change on water and sediment yields. The impact of changes in water and sediment yields on wellbeing is more uncertain, not because we are unable to measure the effect of resulting changes in water prices, but because water prices are poor proxies for the social opportunity cost of the resource. There are limitations in our approach to modeling water and sediment flows. We do not, for example, model the impact of extreme events on sediment flows. Nor do we model the impact of forest thinning on snow cover and sublimation. But the errors associated with these omissions are likely to be small relative to the errors induced by price distortions.

While we do not have a true measure of the social opportunity cost of water in Arizona, we do know that water revenues to the water and power utilities are a poor proxy for this. Adding the cross subsidies from power to water in the SRP accounts partially corrects for this, and indicates that the water supply benefits of forest thinning are potentially significant. The thinning scenario applied in our analysis is different from the 50% basal cover reduction envisaged by 4FRI. It implies rates of thinning of canopy cover that lie between 0% and 50%, and so across the watershed would involve less than 50% reduction in basal cover. It does, however, help to show how sensitive the water supply benefits (and erosion disbenefits) are to increasing levels of canopy reduction. The contribution of the general approach is that we are able to translate the biophysical impacts of changes in vegetation cover into an estimate of the economic cost or benefit to the people of the region. We are also able to identify how much of that would be in direct costs or benefits to water consumers and how much would be in indirect costs or benefits to consumers of other goods and services.

## Supporting Information

S1 File APPENDIX A: Reservoir sedimentation and storage capacity loss. (DOCX)

S2 File APPENDIX B: Hydrological flows. (DOCX)

S3 File APPENDIX C: Groundwater pumping. (DOCX)
