# Supplementary material for: The Impact of Forest Thinning on the Reliability of Water Supply in Central Arizona
Source: PLoS One. 2015 Apr 2;10(4):e0121596. doi: 10.1371/journal.pone.0121596 (PMC4383454; doi:10.1371/journal.pone.0121596)
Supplement: S1 File — (DOCX) [file pone.0121596.s001.docx]

# Appendix A:

# Reservoir sedimentation and storage capacity loss

Modeling reservoir capacity loss from LULC-driven sedimentation processes is an approach that requires several steps of analysis. First we model the impact of land cover on the on-site soil erosion given a set of exogenous variables (i.e. precipitation, slope and soil characteristics). Then, we estimate the percentage of the eroded soil reaching the river network and thus the reservoir. Finally sediment yield expressed in weight must be transformed into volume units according to the specific bulk density of the debris.

## A.1. Soil erosion

We adopt the Universal Soil Loss Equation (USLE) approach [[1](#_ENREF_1)] to estimate soil erosion and subsequently sediment loading in the study area. The USLE is an erosion model designed to predict the longtime average soil losses in runoff from specific field areas in specific cropping and management systems. It only predicts soil loss from sheet-and-rill erosion. As soil erosion is influenced by many different variables, the essence of the USLE is to isolate each variable and reduce its effect to a number, so that when the numbers are multiplied together the answer is the amount of soil loss. The factors that control soil erosion are combined in the following empirical form [[1](#_ENREF_1)]:

$A_{x}=R_{x}*K_{x}*LS_{x}*C_{x}*P_{x}$ (a.1)

where *A_x_* expresses soil loss (t ha^-1^ yr^-1^) for the *x* pixel; *R* represents rain erosivity (MJ mm^-1^/ha h^-1^ yr^-1^; *K* is a measure of soil erodibility (t ha^-1^ h^-1^/ha MJ^-1^ mm^-1^) ; *LS* is slope length and steepness combined in a single index; *C* is an index of land cover practice; and *P* represents soil management practice. The *K* (soil erodibility) and *R* (rainfall) factors are dimensional and estimate the total soil loss from a given soil under the reference base conditions. The *L*, *S*, *C*, and *P* factors are dimensionless percentages ratios, which serve as correction terms to adjust the predicted soil loss to site-specific conditions.

Rain erosivity can be determined from rainfall data. Renard & Freimund [[2](#_ENREF_2)] used regression analysis to establish correlations between measured *R* values and more readily available precipitation data across the US. Their estimated relationships between mean annual precipitation $\Omega$ (mm) and the *R*-factor (MJ mm ha^-1^ h^-1^ year^-1^) are the following:

$R_{x}=0.0483\Omega_{x}^{1.610}$ with $\Omega_{x}<850mm$

(a.2)

$R_{x}=587.8-1.219\Omega_{x}+0.004105\Omega_{x}^{2}$ with $\Omega_{x}\geq850mm$

We adopt these equations for estimating the spatial distribution of the *R* index using as input the 30 arc second mean monthly precipitation maps for the period 1971-2000 provided by the PRISM Climate Group (http://www.prism.oregonstate.edu/). Since only precipitation in form of rainfall is producing an erosion effect on soil, we consider annual precipitation as the sum of the monthly maps net of snowfall. Following Willmott et al. [[3](#_ENREF_3)], we assume that all precipitation in a given month is snowfall if the mean monthly temperature is below the temperature threshold of –1°C.

The spatially distributed *K* factor expressing inherent erodibility of the soil is taken from the 1:250,000-scale U.S. General Soil Map STATSGO Database and converted to metric units according to [[4](#_ENREF_4)]. For the purpose of computing the effect of slope and slope-length, we used the approach developed by [[5](#_ENREF_5)], derived from unit stream power theory, that explicitly uses the hydrology of the up-slope contributing area instead of the upslope length to account for flow convergence and rilling. This concept was then adapted, for computation purposes within GIS, in finite difference form for erosion in a grid cell representing a hillslope segment [[6](#_ENREF_6),[7](#_ENREF_7)]. The Upslope Contributing Area approach for the estimation of LS factor is defined by the following equation [[7](#_ENREF_7)]:

$LS_{x}=\left(1+n\right)\left(\frac{U_{x}}{22.13}\right)^{n}\left(\frac{\sin\theta_{x}}{0.0896}\right)^{1.3}$ (a.3)

where *U_x_* is the specific catchment area, i.e. the upslope contributing area for the *x* pixel per unit width of contour (or rill), expressed in m^2^ m^-1^; $\theta_{x}$is the slope angle in degrees; and *n* is a calibrated value which ranges between 0.4 and 0.6 according to the prevailing type of flow and soil conditions. In implementing eq. (a.3) within GIS, each grid cell is regarded as a slope segment with a uniform slope. The upslope contributing area is estimated by the flow accumulation grid computed as the sum of the cells from which water flows into the cell of interest. The resulting layer was then multiplied by the cell size expressed in meters (30 m), producing a layer, which expresses the *U_x_* value for each *x* pixel. In order to avoid zero values for *LS* at high points - primarily peaks and ridges - without inflowing cells (i.e. without upslope contributing area), we assigned them a value of 0.5 multiplied by cell resolution as in the Non-cumulative slope length (NCSL) approach proposed by [[8](#_ENREF_8)]. This calculation was based on the assumption that the calculations for slope length were from the center of the cell to the center of its input cell. Therefore, as high points do not have an input cell, the 0.5 value represents only the erosion occurring within the half of that cell that is uphill of the center point.

In estimating the *LS* factor a maximum length threshold should be taken into account to appropriately represent the interrill and rill erosion processes since a zone of deposition or concentrated flow would generally occur when the slope becomes long [[9](#_ENREF_9)]. Research indicates that runoff accumulates into a defined channel on most slope lengths less than 122 meters [[10](#_ENREF_10)] and that slope lengths generally do not exceed 300 m [[9](#_ENREF_9)]. The USLE/RUSLE does not apply to channels, so excessive flow lengths were excluded from analysis. We selected the upper bound of 300 m as maximum slope length by creating a mask grid for the *LS* calculation from the flow length raster where values were below this threshold. For the Upslope Contributing Area approach, since the number of inflowing cells depends on the specific map resolution adopted (30x30m), we set a 10 cells upper bound identifying the maximum upslope contributing area related to the maximum slope length.

The land cover factor (*C*) compares the erosion of a bare soil plot with that from a plot with a specific plant cover. For non-vegetated forest cover C-factors are selected referring to the N-SPECT (Nonpoint Source Pollution and Erosion Comparison Tool) technical guide by NOAA (http://www.csc.noaa.gov/digitalcoast/tools/nspect/). For vegetation land cover categories we adopt an exponential functional relationship between C-factors and the Normalized Difference Vegetation Index (NDVI):

$C_{xi}=e^{\left(-\alpha\frac{NDVI_{xi}}{\beta-NDVI_{xi}}\right)}$ (a.4)

where $\alpha$is a calibration term that we set at $\alpha=2.7$, and $\beta<NDVI_{\max}$ approximates the maximum NDVI value in the study area. Since the maximum NDVI value is estimated at 0.68 for sub-alpine forest at 100% canopy cover, we assume $\beta=0.7$. The NDVI values are obtained as averages from remote sensing observations for September-October and May-June periods in 5 different years during 1995-2005. This would control for both inter annual and intra annual variations in vegetation "greenness", thus representing typical annual conditions at a coarse scale during the period.

Mean NDVI values for non-forest vegetation cover categories were then used to estimate mean C-factors applying eq. (a.4). For forest vegetation we overlap the NDVI maps with the percent canopy cover map estimating mean NDVI values at each percent canopy cover deciles. Functional relationships between NDVI and percent canopy cover for each forest category ($L_{x1}$= Subalpine forest, $L_{x2}$= Ponderosa pine, $L_{x3}$= Piñon-Juniper, $L_{x4}$= Deciduous forest) were then derived through curve fitting estimation (Table a.1).

The *P* factor is the support practice factor. It expresses the effects of supporting conservation practices, such as contouring, buffer strips of close-growing vegetation, and terracing on soil loss at a particular site. In the study area we assumed there was no specific soil conservation practice, thus *P_x_* = 1 for all the pixels. Thus, applying eq. (a.1) we obtain a map expressing the spatial distribution of soil erosion across the SRP basin.

## A.2. Sediment delivery

In relatively large watersheds, most sediment gets deposited within the watershed and only a fraction of soil that is eroded from hillslopes will reach the stream system or watershed outlet. This fraction or portion of sediment that is available for delivery is referred to as the Sediment Delivery Ratio (SDR). This ratio is then multiplied by the predicted erosion rate to estimate the percent of eroded sediment to reach the watershed outlet or the stream network.

Different models have been developed to estimate the SDR based respectively on the area power function [[11](#_ENREF_11),[12](#_ENREF_12),[13](#_ENREF_13)], rainfall-runoff factors [[14](#_ENREF_14)], slope gradient [[15](#_ENREF_15)], relief-length ratio [[11](#_ENREF_11)], particle size [[16](#_ENREF_16)], water runoff travel time [[17](#_ENREF_17),[18](#_ENREF_18),[19](#_ENREF_19)], distance-slope [[20](#_ENREF_20)], and runoff curve numbers [[21](#_ENREF_21)].

We adopt the SEDD model proposed by [[18](#_ENREF_18)]. The SEDD model discretizes a watershed into morphological units (areas of defined aspect, length, and steepness) and determines a sediment delivery ratio (SDR) for each unit [[22](#_ENREF_22)]. This model is based on the hypothesis that the sediment production arriving from a sediment source into a stream reach can be transported to the basin outlet. This hypothesis agrees with *Playfair's law* of stream morphology which states that over a long time a stream must essentially transport all sediment delivered to it [[23](#_ENREF_23)]. In other words it is hypothesized that the transport capacity of the river flow is not a limiting factor. In our model, however, we assume this only for the clay and silt fraction of the eroded sediment. For sand we apply the SDR to estimate the fraction of eroded sand sediment reaching the stream network. Then, an additional model for river sand deposition is applied to estimate the amount of sand loading reaching the outlet (see **File S3**).

We assumed the *x* pixel as the morphological unit. Thus *SDR_x_*, the fraction of the gross soil loss from pixel *x* that actually reaches a continuous stream system (for sand) or the watershed outlet (for clay and silt), is estimated following [[18](#_ENREF_18)] as a function of travel time:

$SDR_{x}=\exp\left(-\omega t_{x}\right)$ (a.5)

where *tx* is the runoff travel time (sec) from pixel *x* to the stream network; and *ω* is a basin-specific parameter. The travel time, *tx*, is assumed to increase as the length, *l*, of the hydraulic path increases or as the square root of the slope, *s*, of the hydraulic path decreases. If the flow path from the *x* pixel to the nearest channel traverses N pixels, then the travel time from that pixel is calculated by adding the travel time for each of the j*th* pixels located along the flow path [[24](#_ENREF_24)]:

$t_{x}=\sum_{j=1}^{N} \frac{l_{j}}{\sqrt{s_{j}}}$ (a.6)

where *lj* is the length of segment *j* in the flow path (expressed in meters) and is equal to the length of the side or diagonal of a pixel depending on the flow direction in the pixel; and *sj* is the slope (m/m) of the j*th* pixel along the hydraulic path.

Ferro & Minacapilli [[18](#_ENREF_18)] showed that the sediment delivery relationship is independent of the soil erosion model and that the basin-specific *ω* coefficient can be estimated by the following equation, which depends primarily on watershed morphological data:

$\omega=\frac{\ln\left(SDR_{U}\right)}{1000a}$ (a.7)

where *SDR_U_* is the watershed sediment delivery ratio and *a* is an estimated coefficient depicting the sediment transport efficiency. Ferro [[17](#_ENREF_17)] showed that the *a* coefficient increases linearly with the median travel time (*tm*) of a given basin according to the following equation:

$a=0.001116t_{m}$ (a.8)

In order to estimate the *SDR_U_* of eq. (a.7) we use the area based functional relationship proposed by [[13](#_ENREF_13)]:

$SDR_{U}=0.4724U^{-0.125}$ (a.9)

where *U* is the catchment area (km^2^). The inverse relationship between *SDR_U_* and *U* can be explained by the *upland theory* of Boyce [[23](#_ENREF_23)]. According to this theory the steepest areas of a basin are the main sediment-producing zones, and since average slope decreases with increasing basin size the sediment production per unit area decreases too. We estimated an *SDR_U_* for every dam basin identified in our area of study. Basin median travel time values (*tm*), then *a* coefficients where estimated from the *t_x_* spatial distribution map using eq. (a.8). Following eq. (a.5) we estimated the spatial distribution of the SDR given the current LULC map. Multiplying this ratio by the predicted soil erosion we obtain a map representing the sediment yield from the *x* pixel source reaching the stream network.

## A.3. River sediment transport capacity

Not all the sediment delivered to the stream network is reaching the outlet of the basin. Riverbed deposition depends on the specific sediment transport capacity of each stream link, which is mostly a function of flow velocity and particle size of the sediment. The velocity of flow required to move silt and clay is much smaller than the velocity of flow required to move sand. Since most streamflow exceeds this velocity, silts and clays are not found in appreciable quantities in streambeds. In most cases, these fine materials tend to wash on through the system and are often referred to as wash load. Wash load is carried within the water column as part of the flow, and therefore moves with the mean velocity of the stream. Accordingly, our sediment transport capacity model predicting streambed sediment deposition only considers sand particles, assuming that clay and silt components of the sediment eroded and delivered to the stream network are reaching the outlet of the basin.

Using Yang's [[25](#_ENREF_25)] equation, and average value for Mannings roughness coefficient of 0.025, we predicted sediment transport capacity for sand particles along the river network from:

$TC_{k}=\frac{86\theta_{k}^{1.3}Q_{Lk}^{1.4}}{v\omega_{k}^{0.4}}$ (a.10)

where *TC_x_* represents the sediment transport capacity (t yr^-1^) for sand particles at the *k^th^* pixel of the river network; *Q_Lk_* is the total annual water flow (in million litres) trough the river pixel *k* and estimated by our hydrological model as the sum of water yields from all the *x^th^* pixels in the watershed that are upstream from river pixel *k*; *θ_k_* is the sin of the slope at pixel *k*; *v* is the settling velocity of bedload particles; *ω_k_* is the river width (m) at pixel *k*.

The setting velocity of bedload particles is modelled according to the equation of fall velocity of spherical particles proposed by [[26](#_ENREF_26)]:

(a.11)

where *v* is the settling velocity in m/s; $g=9.81$ m s^-2^ is the acceleration due to gravity; *d* is particle diameter expressed in meters that we assume equal to 0.002; $w=1.004*10^{-6}$ m^2^ s^-1^ is the kinematic viscosity of freshwater at 20°*C*; and *ϕ* represents the relative density as the fraction of the density of sediment particle (assumed at 1.922 t m^-3^ as for wet sand) over the density of water (0.9982 t m^-3^ at 20°*C*). The hydraulic radius of the river flow is estimated using downstream hydraulic geometry [[27](#_ENREF_27)]:

$\omega_{k}=3.004Q_{k}^{0.426}d_{m}^{-0.002}\theta_{k}^{-0.153}$ (a.12)

with *Q_k_* representing water flow (m^3^ s^-1^) at pixel *k*; *d_m_* is the median particle size (m) that for sand we assume equal to 0.002; and *θ_k_* is the slope expressed in m m^-1^. Water flow *Q_k_* is obtained from the annual water yield estimated through our hydrological model. Considering the strongly seasonal pattern of precipitation in the watershed, the flow (m^3^ s^-1^) at the river pixel *k* from its upslope contributing area is obtained by concentrating in a 6-month period the predicted annual yield from the sum of all the pixels flowing into *k*.

After splitting the stream network in stream links, each representing a *b^th^* sub-basin, stream links are ordered according to the Strahler order classification using ArcGis, where an increasing index b=1,2,3,..,*n* is assigned from the most upstream to the most downstream link on the same flow path. Then, following eq. [a.10] we estimate the sand transport capacity ($TC_{bk}$) for each *k^th^* river pixel of the *b^th^* stream link. The mean transport capacity rate for sand ($\varepsilon_{b}$) is estimated for each *b^th^* stream link and related watershed as the ratio between the mean transport capacity ($TC_{b}$) considering the *n_b_* pixels of the stream link and the mean sand delivery ($S_{b}$) to that stream link:

$\varepsilon_{b}(L_{xi})=\frac{TC_{b}(L_{xi})}{S_{b}(L_{xi})}$ (a.13)

with,

$TC_{b}=\frac{\sum_{k}^{n} TC_{bk}}{n_{b}}$ (a.14)

$S_{b}=\frac{\sum_{k}^{n_{b}} S_{bk}}{n_{b}}=\frac{\sum_{k}^{n_{b}} \sum_{x\in b} \upsilon_{sx}^{k}A_{x}^{k}SDR_{x}^{k}}{n_{b}}$ (a.15)

where $\varepsilon_{b}=1$ if $TC_{b}\geq S_{b}$and $0\leq\varepsilon_{b}<1$ otherwise. Mean sand delivery ($S_{b}$) is the average across the *n_b_* pixels of the b*^th^* stream link of the sand sediment delivered to each *k^th^* pixel from the sum of the sand sediment yield from all the *x^th^* inflowing pixels upstream from *k* and within the *b^th^* sub-basin (i.e. $x\in b$). It is therefore a function of the soil erosion $A_{x}(L_{xi})$, sediment delivery ratio to the stream network $(SDR_{x})$, and the percentage of sand ($\upsilon_{sx}$) in superficial soil layer at each *x^th^* pixel. It can be noted that a decrease in percent canopy cover of forest will both increase mean sediment transport capacity $TC_{b}$from increased hydrological flows and increase mean sediment flow $S_{b}$from increased soil erosion. Thus the net marginal effect on the mean transport capacity rate will be uncertain, mostly depending on the spatial distribution of local characteristics (i.e. topography, precipitation, temperature, soil characteristics, distance from the nearest stream, and forest type).

The stream link order is used for routing sediment flows downstream through the stream network after cumulative reduction by the mean transport capacity rates from the upstream links. In other words, taking into account sediment deposition along the flow path, the mean sand delivery for any *b^th^* stream link as expressed in eq. [a.15] becomes:

$S_{b}=\frac{\sum_{k}^{n_{b}} S_{bk}+\varepsilon_{b-1}(S_{b-1}+\varepsilon_{b-2}(S_{b-2}+\varepsilon_{b-3}(S_{b-3}+... \varepsilon_{b-n-1}(S_{b-n-1}+\varepsilon_{b-n}S_{b-n}))))}{n_{b}}$ (a.16)

Using this new expression, mean transport capacity rates for sand are estimated for each stream link on a flow path and then associated to all the sub-basin pixels of the same link.

## A.4. Reservoir sedimentation and storage loss conversion

The contribution (in tons per year) of the *x^th^* watershed pixel to the annual sediment yield of a reservoir at the outlet,$J_{x}$, is estimated as sum of the sand ($J_{sx}$), clay ($J_{cx}$), and silt ($J_{dx}$) sediment:

$J_{x}=J_{sx}+J_{cx}+J_{dx}$ (a.17)

$$J_{sx}=\left(\varepsilon_{b}\varepsilon_{b+1}... \varepsilon_{n}\right)\upsilon_{sx}A_{x}SDR_{x}$$

$$J_{cx}=\upsilon_{cx}A_{x}SDR_{x}$$

$$J_{dx}=\upsilon_{dx}A_{x}SDR_{x}$$

where $\upsilon_{sx},\upsilon_{cx},\upsilon_{dx}$represent respectively the percentages of sand, clay and silt of the soil at pixel *x* and obtained from the U.S. General Soil Map STATSGO Database [[28](#_ENREF_28)]. The sand sediment yield to the reservoir from pixel *x* is obtained by multiplying sand sediment delivery to the river network by the mean transport capacity rate of the *b^th^* stream link identifying the sub-basin containing *x*, and by all the mean transport capacity rates of the *n* stream links along the downstream path to the reservoir.

Sediment yield to the reservoir is then converted from weight to volume following [[29](#_ENREF_29)]:

$W_{x}=\frac{J_{cx}}{J_{x}}W_{c}+\frac{J_{sx}}{J_{x}}W_{s}+\frac{J_{dx}}{J_{x}}W_{d}$ (a.18)

where $W_{x}$is the estimated density of sediment deposits in the reservoir coming from the *x^th^* pixel in the watershed (t/m^3^) and $W_{c},W_{s},W_{d}$express the unit weight of clay, silt and sand respectively that vary according to a set of reservoir operation strategies (Table a.2). We assume Roosevelt reservoir on the Salt River to follow type 1 operational rule and Horseshoe reservoir on the Verde River to follow type 2 operational rule. To simplify the model we do not consider further implications of the compaction time, which modifies unit weight during the lifespan of the reservoir [[30](#_ENREF_30)].

The reciprocal of the sediment density ($1/W_{x}$) is then multiplied by the estimated sediment yield:

$D_{x}=J_{x}\frac{1}{W_{x}}$ [a.19]

where $D_{x}(L_{xi})$ is the annual marginal contribution (m^3^) by the *x^th^* pixel in the watershed to the storage capacity loss of the reservoir.

# References

1. Wischmeier WH, Smith DD (1978) Predicting Rainfall Erosion Losses - A Guide to Conservation Planning. Agricultural Handbook 537. In: Agriculture USDo, editor. 537 ed.

2. Renard KG, Freimund JR (1994) Using monthly precipitation data to estimate the R-factor in the revised USLE. Journal of Hydrology 157: 287-306.

3. Willmott CJ, Rowe CM, Mintz Y (1985) Climatology of the terrestrial seasonal water cycle. Int J Climatol 5: 589-606.

4. Foster GR, McCool DK, Renard KG, Moldenhauer WC (1981) Conversion of the universal soil loss equation to SI metric units. Journal of Soil and Water Conservation 36: 355-359.

5. Moore LD, Burch GJ (1986) Physical Basis of the Length-slope Factor in the Universal Soil Loss Equation. Soil Science Society of America 50: 1294-1298.

6. Desmet PJJ, Govers G (1996) A GIS procedure for automatically calculating the USLE LS factor on topographically complex landscape units. Journal of Soil and Water Conservation 51.

7. Mitasova H, Hofierka J, Zlocha M, Iverson LR (1996) Modelling topographic potential for erosion and deposition using GIS. International Journal of Geographical Information Systems 10: 629-641.

8. Hickey R (2000) Slope angle and slope length solutions for GIS. Cartography 29: 1-8.

9. McCool DK, Foster GR, Weesies GA (1997) Slope lenght and steepness factors (LS). Chapter 4. In: Renard KG, Foster GR, Weesies GA, McCool DK, Yoder DC, editors. Predicting Soil Erosion by Water: A Guide to Conservation Planning with the Revised Universal Soil Loss Equation (RUSLE) Agricultural Handbook 703: U.S. Department of Agriculture.

10. Renard KG, Foster GR, Weesies GA, McCool DK, Yoder DC (1997) Predicting Soil Erosion by Water: A Guide to Conservation Planning with the Revised Universal Soil Loss Equation (USLE). Agricultural Handbook 703. In: Agriculture USDo, editor. 703 ed.

11. Maner SB (1958) Factors affecting sediment delivery rates in the Red Hills physiographic area. Transactions of American Geophysics 39: 669-675.

12. USDA (1983) Sediment sources, yields, and delivery ratios. National Engineering Handbook, Sec 3: Sedimentation. Washington, D.C.: U.S. Department of Agriculture, Natural Resources Conservation Service.

13. Vanoni VA (1975) Sedimentation Engineering, Manual and Report No. 54. New York: American Society of Civil Engineers.

14. Arnold JG, Srinivasan R, Muttiah RS, Williams JR (1998) Large area hydrologic modeling and assessment Part I: Model development. Journal of the American Water Resources Association 34: 73-89.

15. Williams JR, Brendt AD (1972) Sediment yield computed with the Universal Equation. Proceeding of the American Society of Civil Engineers, 98(HY12). 2087-2098 p.

16. Walling DE (1983) The sediment delivery problem. Journal of Hydrology 65: 209-237.

17. Ferro V (1997) Further remarks on a distributed approach to sediment delivery. Hydrological Sciences 42: 633-647.

18. Ferro V, Minacapilli M (1995) Sediment delivery processes at basin scale. Hydrological Sciences 40: 703-717.

19. Ferro V, Porto P, Tusa G (1998) Testing a distributed approach for modelling sediment delivery. Hydrological Sciences 43: 425-442.

20. Sun G, McNulty SG. Modeling soil erosion and transport on forest landscape; 1998 February 16-20, 1998; Reno, Nevada. pp. 187-198.

21. Williams JR (1977) Sediment delivery ratios determined with sediment and runoff models. Journal of International Association of Hydrological Sciences: 168-179.

22. Ferro V, Porto P (2000) Sediment delivery distributed (SEDD) model. Journal of Hydrologic Engineering American Society of Civil Engineers 5: 411-422.

23. Boyce RC (1975) Sediment routing with sediment delivery ratios. Present and prospective technology for predicting sediment yield and sources. Washington, D.C.: U.S. Department of Agriculture. pp. 61-65.

24. Jain MK, Kothyari UC (2000) Estimation of soil erosion and sediment yield using GIS. Hydrological Sciences 45: 771-786.

25. Yang CT (1973) Incipient motion and sediment transport. Journal of the Hydraulics Division, ASCE 99: 1679-1704.

26. Cheng NS (1997) Simplified settling velocity formula for sediment particle. Journal of Hydraulic Engineering 123: 149-152.

27. Lee J-S, Julien PY (2006) Downstream hydraulic geometry of alluvial channels. Journal of Hydraulic Engineering 132: 1347-1352.

28. NRCS (1994) U.S. General Soil Map (STATSGO2). Soil Survey Staff, Natural Resources Conservation Service, U.S. Department of Agriculture.

29. Lara JM, Pemberton EL (1965) Initial Unit Weight of Deposited Sediments. Proceedings, Federal Interagency Sedimentation Conference, 1963. U.S. Agriculture Research Service. 818-845 p.

30. Miller CR (1953) Determination of the Unit Weight of Sediment for Use in Sediment Volume Computations. Denver, Colorado: Bureau of Reclamation.

# Tables

**Table a.1.** USLE land cover C-factor.

| **Land cover type** | **NDVI** | **C-factor** |
| --- | --- | --- |
| Water | - | 0.0000 |
| Developed, open space | - | 0.0130 |
| Developed, low intensity | - | 0.0300 |
| Developed, medium intensity | - | 0.0150 |
| Developed, high intensity | - | 0.0000 |
| Barren land | - | 0.7000 |
| Chaparral | 0.27 | 0.1835 |
| Desert shrub | 0.25 | 0.2231 |
| Subalpine grassland | 0.34 | 0.0781 |
| Plains & Semi-desert grasslands | 0.23 | 0.2668 |
| Transitional | 0.30 | 0.1320 |
| Pasture/hay | 0.51 | 0.0007 |
| Cultivated crops | 0.39 | 0.0335 |
| Woody wetlands | 0.52 | 0.0004 |
| Herbaceous wetlands | 0.53 | 0.0002 |
| Subalpine forest | $NDVI_{x1}=0.511-0.161L_{x1}+0.435L_{x1}^{2}-0.105L_{x1}^{3}$ | $C_{x1}=\exp\left(-\alpha\frac{NDVI_{x1}}{\beta-NDVI_{x1}}\right)$ |
| Ponderosa pine forest | $NDVI_{x2}=0.267+0.7L_{x2}-0.883L_{x2}^{2}+0.588L_{x2}^{3}$ | $C_{x2}=\exp\left(-\alpha\frac{NDVI_{x2}}{\beta-NDVI_{x2}}\right)$ |
| Piñon-Juniper forest | $NDVI_{x3}=0.159+1.02L_{x3}-1.308L_{x3}^{2}+0.753L_{x3}^{3}$ | $C_{x3}=\exp\left(-\alpha\frac{NDVI_{x3}}{\beta-NDVI_{x3}}\right)$ |
| Deciduous forest | $NDVI_{x4}=0.463-0.688L_{x4}+1.931L_{x4}^{2}-1.058L_{x4}^{3}$ | $C_{x4}=\exp\left(-\alpha\frac{NDVI_{x4}}{\beta-NDVI_{x4}}\right)$ |

**Table a.2.** Sediment density coefficients.

| **Operational mode of reservoirs** | **Clay**  **(W_c_)** | **Silt**  **(W_d_)** | **Sand**  **(W_s_)** |
| --- | --- | --- | --- |
| 1. Sediment always or nearly submerged | 0.416 | 1.12 | 1.55 |
| 2. Moderate to considerable reservoir drawdown | 0.561 | 1.14 | 1.55 |
