# Supplementary material for: The Impact of Forest Thinning on the Reliability of Water Supply in Central Arizona
Source: PLoS One. 2015 Apr 2;10(4):e0121596. doi: 10.1371/journal.pone.0121596 (PMC4383454; doi:10.1371/journal.pone.0121596)
Supplement: S2 File — (DOCX) [file pone.0121596.s002.docx]

# Appendix B:

# Hydrological flows

## B.1. Hydrological model

We estimate the spatial distribution of annual water yield across the basin using a water balance approach. This modelling approach has been applied at different scales, ranging from catchment studies to regional and global water balance and climate change scenarios [[1](#_ENREF_1),[2](#_ENREF_2),[3](#_ENREF_3)]. The popularity of this approach in hydrologic studies is most likely due to its simplicity in contrast with complex hydrological models such as SHE [[4](#_ENREF_4)], TOPMODEL [[5](#_ENREF_5)], and SWAT among others. In the Salt and Verde river watershed Ellis et al [[6](#_ENREF_6)] applied the Thornthwaite-Mather climatic water budget (CWB) model [[7](#_ENREF_7)] for modelling the impact of climate change scenarios on water yield. Their model however does not include a variable accounting for the marginal impact of land use and land cover across the basin. When the hydrological effect of land cover is accounted for [[8](#_ENREF_8),[9](#_ENREF_9)], it only depicts discrete variability referring to a limited number of vegetation types, generally ignoring spatial variation within the same vegetation type. This limitation is a common feature in most water budget models. Our approach, on the other hand, focuses on the marginal impact of continuous attributes such as the spatial distribution of percent canopy cover of forest. We estimate annual water yield from the *x^th^* pixel as a function of the *i^th^* land cover, and for forest pixels as a function of percentage canopy cover under the *i^th^* forest type, according to the following expression:

$Y_{xi}=R_{x}-I_{xi}(L_{xi})-E_{xi}(L_{xi})$ (b.1)

where *R_x_* is annual rainfall (mm) on pixel *x*, *I_xi_* is annual water interception loss (mm) by the canopy of the i*th* vegetation type on pixel *x*, and *E_xi_* is actual evapotranspiration (mm). In our hydrological model, the land cover variable $(L_{xi})$ is continuous in space only for forest vegetation, representing percent canopy.

## B.2. Canopy interception loss

Canopy interception loss was estimated adapting the exponential relation proposed by Aston [[10](#_ENREF_10)] to mean daily precipitation:

$I_{xi}=n_{x}S_{xi}\left(1-e^{-k_{xi}R_{x}/S_{xi}}\right)$ (b.2)

where *I_xi_* is the annual interception loss (mm) for pixel *x* under land cover type *i*; *R_x_* is the mean daily rainfall intensity (mm) considering only rainy days in the year ; *k_xi_* is a correction factor for canopy openness that is specific to the land cover type *i*; *S_xi_* is the vegetation-specific maximum canopy storage capacity (mm); and *n_x_* is the number of rainy days during the year on pixel *x*. The parameter $k_{xi}$ equals the fraction of rainfall that falls on the canopy and is the inverse of the canopy openness or free throughfall coefficient. Theoretically, $k_{xi}$ approaches 1 for very dense and closed canopies when all rainfall is used to fill up the canopy storage. In reality, the value of $k_{xi}$ depends on the Leaf Area Index (LAI) but also on such variables as the canopy structure, rainfall intensity, and wind activity. For forest pixels we use the percent canopy cover $(L_{xi})$ as proxy for $k_{xi}$, while for non-forest vegetation and crops, $k_{xi}$ was obtained using mean LAI values for each i*th* vegetation type and applying the relationship proposed by de Jong & Jetten [[11](#_ENREF_11)]:

$k_{i}=0.065LAI_{i}$ (b.3)

Mean LAI values were derived from published functional relationships between NDVI and LAI applicable to coniferous forest [[12](#_ENREF_12)] and non-forest vegetation in drylands [[13](#_ENREF_13)] respectively:

$LAI_{xi}=\left[-\ln\left(\frac{NDVI_{xi}-0.9}{0.01925-0.9}\right)\right]/0.416$ (b.4)

$LAI_{xi}=-0.0897+1.424NDVI_{xi}$ (b.5)

For forest vegetation we use eq. (b.4) to estimate mean LAI at percent canopy cover deciles given the relationship between NDVI and percent canopy cover from Table a.1. Functional relationships between LAI and percent canopy cover for each forest category ($L_{x1}$= Subalpine forest, $L_{x2}$= Ponderosa pine, $L_{x3}$= Piñon-Juniper, $L_{x4}$= Deciduous forest) were then derived through curve fitting estimation (Table b.1). For non-forest vegetation mean LAI values (Table b.1) were derived from eq. (b.5) using remote-sensed mean NDVI values reported in Table a.1 (see File S1).

Canopy storage capacity for subalpine forest was estimated as the average value between S=2.8 reported for Sitka spruce [[14](#_ENREF_14)] and S=3.3 for Douglas-fir [[15](#_ENREF_15)]. Value for piñon-juniper forest was taken from Link et al. (2004). Values for ponderosa pine and deciduous forest were taken as the respective average of reported values referring to US sites [[16](#_ENREF_16)]. The value for deciduous-conifer mixed forest was estimated as the average between ponderosa pine and deciduous forest. Published functional relationships for non-forest vegetation and cultivated crops/pasture were used for estimating canopy storage capacity, respectively [[11](#_ENREF_11)]:

$S_{xi}=0.3063LAI_{xi}+0.5753$ (b.6)

$S_{xi}=0.935+0.498LAI_{xi}-0.00575LAI_{xi}^{2}$ (b.7)

The spatial distribution of the average number of rainy days in a year (*n_x_*) and the mean daily rainfall intensity (*R_x_*) for the rainy days in the year were calculated as 11-year average from daily data available for the period 1995-2005 from 11 Snotel meterological stations in the basin. Mean daily rainfall intensity was regressed against mean annual precipitation (*P*) for the period 1995-2005 for each station obtaining the following exponential relationship:

$R_{x}=5.368+0.014P_{x}-0.003Elev_{x}$ $(R^{2}=.943, n=11)$ (b.8)

The function was then used to estimate the spatial distribution of mean daily rainfall intensity (*R_xt_*) using the annual precipitation map for the period 1995-2005 from the PRISM dataset. The number of rainy days in a year (*n_xt_*) at each *xth* pixel was then obtained by overlaying annual precipitation and daily rainfall intensity maps.

**B.3. Evapotranspiration**

Mean annual evapotranspiration for each pixel *x*, in terms of actual evapotranspiration (*E_x_*), was estimated from potential evapotranspiration (*E_0x_*) and reduction coefficients accounting for plant transpiration (*k_xi_*) and soil evaporation (*s_xi_*) respectively:

$E_{xi}=\left(k_{xi}+s_{xi}\right)E_{0x}, 0\leq\left(k_{xi}+s_{x}\right)\leq1$ (b.9)

Potential evapotranspiration is derived as the sum of long-term averages of monthly *E_0_* (mm) following Hamon's [[17](#_ENREF_17)] equation:

$E_{0x}=\sum_{t=1}^{12} 13.97d_{t}D_{t}^{2}W_{t}$ (b.10)

where *d_t_* is the number of days in month *t*, *D_t_* is the mean monthly hours of daylight in units of 12 h for month *t*, and *W_xt_* is a monthly-average saturated water vapour density term, calculated from mean monthly temperature (*T_xt_*) in degree Celsius [[18](#_ENREF_18)]:

$W_{xt}=0.0495e^{0.062T_{xt}}$ (b.11)

with the spatial distribution of monthly temperature for each x*th* pixel derived from long-term averages of monthly temperature maps (PRISM dataset).

Following Neitsch et al. [[19](#_ENREF_19)], plant transpiration reduction term is obtained from the leaf area index coefficient at each pixel and a reference LAI value ($\alpha$) for dense forest:

$k_{xi}=\frac{LAI_{xi}}{\alpha} 0\leq k_{xi}\leq1$ (b.12)

For non-forest vegetation type we used mean LAI values while for forest we apply the estimated relationship between LAI and percent canopy cover (Table b.1). According to eq. (b.12), when $LAI\geq\alpha$ as for dense forest areas, plant evaporation is equivalent to *E_0_* (i.e. $k_{xi}=1$). On the other hand, the value for $k_{xi}$ should be reduced when plant density and/or leaf area are lower than for full cover conditions (usually at LAI ≥ 3). Then, the plant transpiration reduction term is estimated assuming $\alpha=3$ [[19](#_ENREF_19)].

Soil evaporation reduction coefficient is estimated as residual of plant transpiration adapting an expression (first left term) representing soil evaporation demand relative to the distance from the surface [[19](#_ENREF_19)] and adjusting it for the depth of the soil evaporating layer relative to potential evapotranspiration:

$s_{xi}=\frac{z_{x}}{z_{x}+\exp\left(2.374-0.00713z_{x}\right)}\frac{Z}{E_{0x}}\left(1-k_{xi}\right)$ (b.13)

where *Z* is the maximum depth of the soil evaporating layer that we use as calibration coefficient in our model assuming Z = 295 mm, and *z_x_* is the depth of the soil restrictive layer obtained from the STATSGO National Soil Dataset maps.

# References

1. Alley WM (1984) On the treatment of evapotranspiration, soil moisture accounting, and aquifer recharge in monthly water balance models. Water Resources Research 20: 1137-1149.

2. Mintz Y, Serafini YV (1992) A global monthly climatology of soil moisture and water balance. Climate Dynamics 8: 13-27.

3. Brown TC, Hobbins MT, Ramirez JA (2008) Spatial distribution of water supply in the coterminous United States. Journal of the American Water Resources Association 44: 1474-1487.

4. Abbott MB, Bathurst JC, Cunge JA, O'Connell PE, Rasmussen J (1986) An introduction to the European Hydrological System - Systeme Hydrologique European `SHE´ 1: History and philosophy of a physically-based distributed modeling system. Journal of Hydrology 87: 45-59.

5. Beven KJ, Kirkby MJ (1979) A physically-based variable contributing area model of basin hydrology. Hydrol Sci Bull 24: 43-69.

6. Ellis AW, Hawkins TW, Balling Jr. RC, Gober P (2008) Estimating future runoff levels for a semi-arid fluvial system in central Arizona, USA. Climate Research 35: 227-239.

7. Thornthwaite CW, Mather JR (1955) The water balance. Centerton, NJ: Laboratory of Climatology.

8. Simonit S, Perrings C (2013) Bundling ecosystem services in the Panama Canal watershed. Proceedings of the National Academy of Sciences 110: 9326-9331.

9. Tallis H, Polasky S (2009) Mapping and valuing ecosystem services as an approach for conservation and natural-resource management. Annals of the New York Academy of Sciences 1162: 265-283.

10. Aston AR (1979) Rainfall interception by eight small trees. Journal of Hydrology 42: 383-396.

11. de Jong SM, Jetten VG (2007) Estimating spatial patterns of rainfall interception from remotely sensed vegetation indices and spectral mixture analysis. International Journal of Geographical Information Science 21: 529-545.

12. Gong P, Pu R, Miller JR (1995) Coniferous forest leaf area index estimation along the Oregon transect using compact airborne spectrographic imager data. Photogrammetric Engineering and Remote Sensing 61: 1107-1117.

13. Fan L, Gao Y, Bruck H, Bernhofer C (2009) Investigating the relationship between NDVI and LAI in semi-arid grassland in Inner Mongolia using in-situ measurements. Theoretical and Applied Climatology 95: 151-156.

14. Hutchings NJ, Milne R, Crowther JM (1988) Canopy storage capacity and its vertical distribution in a Sitka spruce canopy. Journal of Hydrology 104: 161-171.

15. Pypker TG, Bond BJ, Link TE, Marks D, Unsworth MH (2005) The importance of canopy sructure in controlling the interception loss of rainfall: Examples from young and old-growth Douglas-fir forest. Agricultural and Forest Meteorology 130: 113-129.

16. Breuer L, Eckhardt K, Frede H-G (2003) Plant parameter values for models in temperate climates. Ecological Modelling 169: 237-293.

17. Hamon WR (1961) Estimating potential evapotranspiration. Proceedings of the American Society of Civil Engineers 871: 107-120.

18. Wolock DM, McCabe GJ (1999) Explaining spatial variability in mean annual runoff in the coterminous United States. Climate Research 11: 149-159.

19. Neitsch SL, Arnold JG, Kiniry JR, Williams JR, King KW (2005) Soil and water assessment tool (SWAT): theoretical documentation, version 2005. College Station, Texas: Texas Water Resources Institute.

# Tables

**Table b.1.** Leaf area index (LAI) and canopy interception factors.

| **LULC** | **LAI** | **k** | **S** |
| --- | --- | --- | --- |
| Subalpine forest | $LAI_{x1}=1.903-0.421L_{x1}+1.081L_{x1}^{2}+0.763L_{x1}^{3}$ | $0.2\leq L_{x1}\leq1$ | 3.00 |
| Ponderosa pine forest | $LAI_{x2}=0.513+5.016L_{x2}-8.123L_{x2}^{2}+5.836L_{x2}^{3}$ | $0.2\leq L_{x2}\leq1$ | 2.20 |
| Piñon-Juniper forest | $LAI_{x3}=0.151+5.588L_{x3}-8.293L_{x3}^{2}+5.338L_{x3}^{3}$ | $0.2\leq L_{x3}\leq1$ | 2.00 |
| Deciduous-conifer mixed forest | $LAI_{x4}=1.892-4.953L_{x4}+12.429L_{x4}^{2}-6.344L_{x4}^{3}$ | $0.2\leq L_{x4}\leq1$ | 1.65 |
| Chaparral | 0.29 | 0.019 | 0.66 |
| Desert shrub | 0.27 | 0.018 | 0.66 |
| Subalpine grassland | 0.39 | 0.025 | 0.69 |
| Plains & Semi-desert grasslands | 0.24 | 0.016 | 0.65 |
| Transitional | 0.34 | 0.022 | 0.68 |
| Pasture/hay | 0.64 | 0.042 | 0.77 |
| Cultivated crops | 0.47 | 0.031 | 1.17 |
| Woody wetlands | 0.65 | 0.042 | 0.77 |
| Herbaceous wetlands | 0.67 | 0.044 | 0.78 |
