# Supplementary material for: The Impact of Forest Thinning on the Reliability of Water Supply in Central Arizona
Source: PLoS One. 2015 Apr 2;10(4):e0121596. doi: 10.1371/journal.pone.0121596 (PMC4383454; doi:10.1371/journal.pone.0121596)
Supplement: S3 File — (DOCX) [file pone.0121596.s003.docx]

# Appendix C:

# Groundwater pumping

We model groundwater pumping dynamics using the Theis equation [[1](#_ENREF_1),[2](#_ENREF_2)]. This equation is commonly used for drawdown estimation generated by well pumping in an unconfined aquifer under transient conditions. Drawdown is the drop in level of water in a well when water is being pumped and it measures the difference (in feet or meters) between the static level and the pumping level. Drawdown is a function of the cone of depression, which decreases with distance from the well and progressively increases with time. Under the assumption that the aquifer reaches a new steady-state condition within a year, we do not consider the time variable included in the original equation (i.e. *t*=1). At the steady state, the cone of depression has spread until it has stopped an equal amount of water leaving the system and the additional drawdown from the abstraction well reaches its maximum. Since often the pumping wells are at a close distance from each other, multiple cones of depression can intersect and result in greater drawdown than expected from a single well. As the equation governing groundwater flow is linear, the drawdown at any point is found by summing the drawdown produced by all the wells as their cones of depression intersect [[3](#_ENREF_3)].

Thus, the drawdown *h_ij_* at the *j^th^* well identified by the *i^th^* pixel is the sum of the drawdown generated by that well and by all the other wells whose respective cones of depression intersect at pixel *i*:

$h_{ij}=\sum_{j=1}^{n} d_{ij}\left(r_{ij},Q_{j}\right)$ (c.1)

with

$d_{ijt}=\frac{Q_{jt}}{4\pi T_{i}}\left[\ln\left(\frac{4T_{i}}{r_{ij}^{2}S_{i}}\right)-0.5772\right]$ (c.2)

where $h_{ij}$ represents a measure of drawdown (m) at pixel *i* given the radial distance $r_{ij}$(m) from well *j*;$Q_{j}$is the pumping rate (m^3^/year) of the *j^th^* well; $\pi$is the constant 3.14... ; *T_i_* is the transmissivity of the confined aquifer at pixel *i* expressed in m^2^/year; and *S_i_* is the dimensionless storativity index at pixel *i*. Digitalized maps for the transmissivity and specific yield of the aquifer in the area of study are provided by the Arizona Department of Water Resources Regional Groundwater Flow Model [[4](#_ENREF_4)]. As specific yield govern the behavior of unconfined aquifers over longer timescales, we can use it as a proxy for storativity under steady-state conditions [[5](#_ENREF_5)]. Since the aquifer modeled by the ADWR in the Phoenix metropolitan area is a multi-layered aquifer system consisting of three aquifers, each with its own hydraulic characteristics but separated by interfaces that allow unrestricted crossflow, the response to pumping will be analogous to that of a single-layered aquifer whose transmissivity and storativity are equal to the sum of the transmissivity and storativity of the individual layers [[6](#_ENREF_6)].

# References

1. Cooper HH, Jacob CE (1946) A generalized graphical method for evaluating formation constants and summarizing well field history. Am Geophys Union Trans 27: 526-534.

2. Theis CV (1935) The relation between the lowering of the piezometric surface and the rate and duration of discharge of a well using ground-water storage. Transactions American Geophysical Union 16: 519-524.

3. Brozovic N, Sunding DL, Zilberman D (2010) On the spatial nature of the groundwater pumping externality. Resource and Energy Economics 32: 154-164.

4. ADWR (2009) Regional groundwater flow model of the Salt River valley. Phoenix Active Management Area. Model update and calibration. Arizona Department of Water Resources.

5. Neuman SP (1972) Theory of flow in unconfined aquifers considering delayed response of the water table. Water Resources Research 8: 1031-1044.

6. Kruseman GP, De Ridder NA (2000) Analysis and Evaluation of Pumping Test Data. Wageningen, The Netherlands: International Institute for Land Reclamation and Improvement.
